# Multiomics analysis of cassava with different ploidy levels in response to*Tetranychus cinnabarinus*

**DOI:** 10.1186/s12870-025-07522-6

**Published:** 2025-11-07

**Authors:** Wanling Wei, Yuanhang Huang, Zhenling Huang, Haixia Yang, Zhaoqin Cai, Ruolan Huang, Wen He, Huixian Chen, Zhenhua Liang, Lixia Ruan, Xiu Lan, Qingwen Deng, Guanyong He, Qing Chen, Jinren Luo, Maogui Wei, Hengrui Li

**Affiliations:** 1 Guangxi South Subtropical Agricultural Sciences Research Institute, Chongzuo, 532415 China; 2 Guangxi Subtropical Crops Research Institute, Nanning, 530001 China; 3 College of Agriculture, Guangxi University, Nanning, 530004 China; 4 Environment and Plant Protection Institute, Chinese Academy of Tropical Agricultural Sciences, Haikou, 571101 China; 5 Guangxi Tianyuan Biochemical Co., Ltd, Nanning, 530007 China

**Keywords:** Cassava, Autotetraploid, Biotic stress, *Tetranychus cinnabarinus*, Amino acids

## Abstract

**Background:**

Cassava (*Manihot esculenta* Crantz) is cultivated for its starchy root and mainly used as starch and biofuel feedstock in China. The red spider mite (*Tetranychus cinnabarinus* Boisduval) is one of the main insect pests reducing cassava yields and becoming more and more serious with regard to the increasing continuous cropping years in China.

**Results:**

The results indicated that SC205 (4×) was more resistant to *T. cinnabarinus* infestation than SC205 (2×) according to the leaf damage ingestion, nutrient substance and secondary metabolite results. The *T. cinnabarinus* infestation triggered the expression of many genes and various metabolic processes reaction. Under the mite feeding stress, SC205 (2×) and SC205 (4×) shared 4494 and 5849 differentially expressed genes (DEGs) at 2 and 8 days, respectively. The DEGs were found enriched in the defense pathways flavonoid biosynthesis (map00941) and the flavone and flavonol biosynthesis (map00944), while, differentially accumulated metabolites (DAMs) were also found enhanced in flavonol biosynthesis (map00944) and phenylpropanoid biosynthesis pathway (map00940). Integrative analysis revealed that under 8-day pest hazards, both DEGs and DAMs in SC205 (2×) and SC205 (4×) were significantly co-enriched in several key pathways, including alpha-linolenic acid metabolism, ABC transporters, galactose metabolism, ascorbate and aldarate metabolism, alanine, aspartate and glutamate metabolism, and tyrosine metabolism. These findings suggest that carbohydrate metabolism and amino acid metabolism play crucial roles in cassava’s resistance to *T. cinnabarinus* infection.

**Conclusions:**

Our study reveal the mechanisms of how cassava diploid and its autopolyploid in response to the feeding of *T. cinnabarinus* and provides data support for the precise analysis of cassava resistance and mite resistance breeding in further research.

**Supplementary Information:**

The online version contains supplementary material available at 10.1186/s12870-025-07522-6.

## Introduction

Cassava (*Manihot esculenta* Crantz) is widely cultivated for its starchy roots in tropical and subtropical areas, which is used as staple food for nearly one billion people in 105 countries [1–3]. Cassava is also used as starch and biofuel feedstock [4, 5]. *Tetranychus cinnabarinus* is one of the damaged spider mites and has been reported in most of the crop planting areas, which was known as worldwide inset pest in cassava production [6]. *T. cinnabarinus* appear rapidly during summer, especially when drought occurred. They infest cassava from the bottom leaves upwards by piercing the parenchyma cells using stylets to suck nutrients, leading to severe chlorosis or even fall off of cassava leaves [7]. When the population density of *T. cinnabarinus* ranges from 200–1000/leaf in almost all leaves, it could bring up to 50–70% reduction or even no harvest in cassava production [8, 9]. Furthermore, water deficient stress could be increased with the global warming due to augmentation in evapotranspiration amounts. In the mean while, cassava was proved as the least affected crop to future climatic changes when compared with other crops such as maize, potato, beans, sorghum, banana, millets [10, 11]. The damage caused by *T. cinnabarinus* become more and more serious with regard to both the increasing drought stress and planting area of cassava in the future, threatening the food security throughout the developing world. Therefore, enhancing the mite resistance of cassava has become one of the important issues worldwile that need to be addressed.

Polyploidy, which was the presence of more than two chromosome sets within the cells of an organism, occurs via the duplication of single or combined differentiated genomes [12–14]. The genome duplication generates several changes at the epigenetic level resulting in altered gene expression [15, 16]. Polyploids were distinguished from diploids by better photosynthetic properties and higher biomass productions and secondary metabolites contents, showing a higher tolerance to biotic and abiotic stresses [17, 18]. In the genus *Leucanthemum*, resistance to the specialist root herbivore *Dichrorampha aeratana* consistently increases with increasing plant ploidy level [19]. Hexaploid wheat and octoploid triticale showed higher photosynthetic capacities and better antioxidant systems [20]. Autotetraploids rice [21]and ryegrass [22] were more drought-tolerant than diploids. Cassava polyploid breeding has drastically improved cassava yield and its tolerance to abiotic stress [23–25]. Tetraploidy induced limited changes in the leaf transcriptomes of cassava between diploid and autotetraploid plants, while the differentially expressed genes (DEGs), especially those upregulated in autotetraploid plants, were strongly associated with hormonal and stress responses [25]. By identification of lncRNAs of the autotetraploid cassava, LNC_001148 and LNC_000160 were found mediate drought tolerance by regulating stomatal density, potentiating drought tolerance in cassava [24]. In comparison to parents, the sexual tetraploid plant of cassava manifest as enlarged biomass, yield, early starch filling, deep colored petiole and root epidermis, and show stronger ability to resist to adversity stresses, owing to its enriched flavonoid biosynthesis and glycolysis/gluconeogenesis [26].

By the resistance evaluation of *T. cinnabarinus* for 531 cassava germplasm found that the resistance of polyploids such as varieties SC205, SC124, and SC6068 to spider mites was higher than that of their diploids, indicating that cassava autotetraploids have good potential for mite resistance [27]. In our previous study, comparison of morphological, physiological and mite resistance characteristics of cassava diploid and its autotetraploid plant revealed that the mite resistance of cassava autotetraploid was stronger than its diploid [28]. However, the resistance mechanisms of tetraploid cassava in response to *T. cinnabarinus* still unknown. In the current study, cassava diploid and its autotetraploid, SC205 (2×) and SC205 (4×), were used as materials. Transcriptome and metabolome analyses were applied to reveal the mechanisms of how cassava diploid and its autopolyploid in response to the feeding of *T.* cinnabarinus.

## Materials and methods

### Plant and inset materials

The cassava variety South China 205 was tested as a diploid parent [SC205 (2×)], which is one of the most popular varieties in Guangxi, China, and provided by the Guangxi South Asian Tropical Agricultural Science Research Institute (GSATAS). Its autotetraploid [SC205 (4×)] was obtained by Economic Crops Research Institute of Guangxi Academy of Agricultural Sciences using colchicine doubling method. After morphological identification and chromosome counting, it was determined to be tetraploid and provided for preservation in the cassava germplasm resource garden of GSATAS in Longzhou county in Guangxi. Leaf lobes of SC205 (4×) are shorter, wider and thicker than that of the diploid, while the green color of autotetraploid leaves also darker than the diploid. *T.* cinnabarinus comes from an indoor subculture population and were feeding at temperature 28 ± 2 ℃, relative humidity (75 ± 5)%, and photoperiod 14 (L): 10 (D). Female adult mites were then selected with the same developmental period and size for subsequent experiments.

### Spider mites’ feeding, assessment of the degree of pest hazards and sampling

The experiment was conducted in a greenhouse of the Guangxi South Asian Tropical Agricultural Science Research Institute (22°20′16″ N, 106°47′ 19″ E). Cassava was planted in plastic pots (height: 30 cm, width: 35 cm) with a mixture of garden soil: peat soil: perlite with a 1:1:1 ratio. The cassava cutting of each genotype were cultivated and well-watered after planting. Forty-five healthy plants of each genotype were chosen for inoculation with cinnabar leaf mite. The 4^th^, 5^th^ and 6^th^ fully unfolded leaves from the top of each cassava plant were marked. Fifteen plants were randomly chosen for the eight-day mites' feeding treatment, which was first conducted on the 60-day after planting(DAP). Sixty mites, 20 mites per leaf, were inoculated on the back of the marked leaves of each plant. Another fifteen plants were randomly selected for the two-day mites’ feeding treatment on the 66 DAP, while the remaining plants were treated as CK and marked as the zero-day mites’ feeding. To prevent the escape of mites, the cassava petiole was immediately wrap with cotton wool soaked in glycerol after the inoculation.

Assessment of the degree of pest hazards and sampling were operated on the 68 DAP. According to the Chinese standard NY/T2445-2013, the degree of pest hazards are separated in to five levels, i.e. 0 (no damage), level 1 (the damage spot area less than 25% of the leaf area), level 2 (the damage spot area range to 26%~50% of the leaf area), level 3 (the damage spot area range to 51%~75% of the leaf area), and level 4 (the damage spot area larger than 76% of the leaf area) [28]. The *T.cinnabarinus* were removed after the assessment of the degree of pest hazards. There were three replicates for each treatment, while five plants with 15 marked-leaves for each replicate were sampled. In total, 18 samples were collected, well-mixed, frozen with liquid nitrogen, and preserved at −80 ℃ for physiological indicators, transcriptome, metabolome, and qRT-PCR measurements.

### Determination of contents of total phenolic, soluble sugar and amino acid of cassava leaves

In our previous studies, total phenols were found as one of the major secondary metabolites in cassava, which varied before and after feeding stress by *T. cinnabarinus* [28], while contents of total phenolic and amino acids were significantly correlated with cassava mite damage index. Soluble sugars in plant play important roles for regulating osmotic pressure and transmit signals in response to biological and abiotic stresses. Thus, we analyzed all the mentioned parameters for directly confirming the *T. cinnabarinus* feeding stress levels of each treatment. The contents of total phenolic, soluble sugar and amino acid of cassava leaves were measured using assay kits (Wuhan Eddie Anti-biotechnology Co., Ltd., Wuhan, China) according to the manufacturer’s instructions, respectively. Three technical replicates were operated for each sample.

### Library preparation and transcriptome sequencing

Total RNA was extracted using TRIzol^®^ Reagent according to the manufacturer’s instructions. Three technical replicates for RNA extraction were operated for each sample and the extracted RNAs were then mixed. RNA quality was then determined by 5300 Bioanalyser (Agilent) and quantified using the ND-2000 (NanoDrop Technologies). Only the high-quality RNA samples (OD260/280 = 1.8 ~ 2.2, OD260/230 ≥ 2.0, RIN ≥ 6.5,28S:18S ≥ 1.0, > 1µg) were used to construct the sequencing library. RNA purification, reverse transcription, library construction and sequencing were performed at Shanghai Majorbio Bio-pharm Biotechnology Co., Ltd. (Shanghai, China) according to the manufacturer’s instructions (Illumina, San Diego, CA). The RNA-seq transcriptome library was prepared following Illumina^®^ Stranded mRNA Prep, Ligation from Illumina (San Diego, CA) using 1µg of total RNA. Shortly, messenger RNA was isolated according to polyA selection method by oligo (dT) beads and then fragmented by fragmentation buffer. Double-stranded cDNA was synthesized using a SuperScript double-stranded cDNA synthesis kit (Invitrogen, CA) with random hexamer primers (Illumina). Then the synthesized cDNA was subjected to end-repair, phosphorylation and ‘A’ base addition according to Illumina’s library construction protocol. Libraries were size selected for cDNA target fragments of 300 bp on 2% Low Range Ultra Agarose followed by PCR amplified using Phusion DNA polymerase (NEB) for 15 PCR cycles. After quantified by Qubit 4.0, paired-end RNA-seq sequencing library was sequenced with the NovaSeq 6000 sequencer (2 × 150 bp read length).

The raw paired end reads were trimmed and quality controlled by fastp [29] with default parameters. Then clean reads were separately aligned to the reference genome with orientation mode using HISAT2 [30] software. The mapped reads of each sample were assembled by StringTie [31] in a reference-based approach. The reference genome assembly employed was Manihot_esculenta_v_GCF_001659605.2 (https://www.ncbi.nlm.nih.gov/assembly/GCF_001659605.2/).

To identify DEGs between two different samples, the expression level of each transcript was calculated according to the transcripts per million reads (TPM) method. RSEM [32] was used to quantify gene abundances. Essentially, differential expression analysis was performed using the DESeq2. DEGs with |log2FC| ≥ 1 and FDR<0.05 (DESeq2) were considered as DEGs. In addition, functional-enrichment and functional-annotation analysis, including Gene Ontology (GO) (http://www.geneontology.org/) and Kyoto Encyclopedia of Genes and Genomes (KEGG) analyses (http://www.genome.jp/kegg/), were performed to obtain the GO and KEGG annotations of DEGs in the reference genome. GO functional enrichment and KEGG pathway analysis were carried out by Goatools and Python scipy, respectively.

### Metabolite profiling and data analysis

For each sample, 50 mg well-mixed sample was added to a 2 mL centrifuge tube and a 6 mm diameter grinding bead was added, while 400 µL of extraction solution (methanol: water = 4:1 (v: v)) containing 0.02 mg/mL of internal standard (L-2-chlorophenylalanine) was used for metabolite extraction. Samples were ground by the Wonbio-96c (Shanghai wanbo biotechnology co., LTD) frozen tissue grinder for 6 min (−10 °C, 50 Hz), followed by low-temperature ultrasonic extraction for 30 min (5 °C, 40 kHz). The samples were preserved at −20 °C for 30 min, centrifuged for 15 min (4 °C, 13000 g), and the supernatant was transferred to the injection vial for LC-MS/MS analysis. As a part of the system conditioning and quality control process, a pooled quality control sample (QC) was prepared by mixing equal volume of all samples. The QC samples were disposed and tested in the same manner as the analytic samples. It helped to represent the whole sample set, which would be injected at regular intervals (every 5–15 samples) in order to monitor the stability of the analysis.

The LC-MS/MS analysis was conducted using the Thermo UHPLC-Q Exactive HF-X system equipped with an ACQUITYHSS T3 column (100 mm × 2.1 mm i.d., 1.8 μm; Waters, USA) by Majorbio Bio-Pharm Technology Co. Ltd. (Shanghai, China). The mobile phases consisted of 0.1% formic acid in water: acetonitrile (95:5, v/v) (solvent A) and 0.1% formic acid in acetonitrile: isopropanol: water (47.5: 47.5:5, v/v/v) (solvent B). The solvent gradient used was: 0%–20% (B), 0–3 min; 20%–35% (B), 3–4.5 min; 35%–100% (B), 4.5–5 min; 100% (B), 5–6.3 min; 100% −0%(B), 6.3–6.4 min; 0% (B), 6.4–8 min. The flow rate was 0.40 mL/min and the column temperature was 40℃. The mass spectrometric data were collected using an electrospray ionization (ESI) source operating in positive mode and negative mode. The optimal conditions were set as followed: source temperature at 425℃, sheath gas flow rate at 50 ar, aux gas flow rate at 13 arb, ion-spray voltage floating (ISVF) at −3500 V in negative mode and 3500 V in positive mode, respectively, normalized collision energy, 20–40-60 V rolling for MS/MS. Full MS resolution was 60,000, while MS/MS resolution was 7500. Data acquisition was performed with the Data Dependent Acquisition (DDA) mode. The detection was carried out over a mass range of 70–1050 m/z.

### Quantitative real-time PCR (qRT-PCR)

Total RNA was extracted from diploid and autotetraploid cassava leaf samples using the FastPure Universal Plant Total RNA Isolation Kit (Vazyme, Beijing, China). The purity, concentration, and integrity of total RNA were measured by a BioTek Epoch full wavelength ELISA reader (BioTek, Beijing, China) and electrophoresis apparatus (Beijing Liuyi Biotechnology Co., Ltd, Beijing, China). The synthesis of first-strand cDNA was performed using the HiScript II Q RT SuperMix for qPCR (+ gDNA wiper) (Vazyme, Dalian, China). qRT-PCR was carried out in a 20 µL reaction volume using ChamQ Universal SYBR qPCR Master Mix Kit (Vazyme, Beijing, China) and a qTOWER2.2 Real-Time System (Analytik Jena, Inc., Germany). The NCBI-Primer-BLAST tool was used to design the primers (Table A1). The relative gene expression level was calculated using the 2^− ΔΔCT^ method.

### Integrative analysis of transcriptome and metabolome

DEGs and MADs were co-mapped to the KEGG pathway database. Pearson correlation coefficients and their corresponding *p*-values were used to identify metabolites and related genes within the significantly altered KEGG metabolic pathways, based on which pathway maps were constructed.

### Statistical analysis

One-way analysis of variance (ANOVA) and Duncan's multiple range test (*P* < 0.05) were applied for statistical analysis using SPSS 25.0 Statistics (SPSS Inc., Chicago, IL, USA).

## Results

### Phenotype response of the diploid and autotetraploid cassava under *T. cinnabarinus* stress

**Fig. 1:**
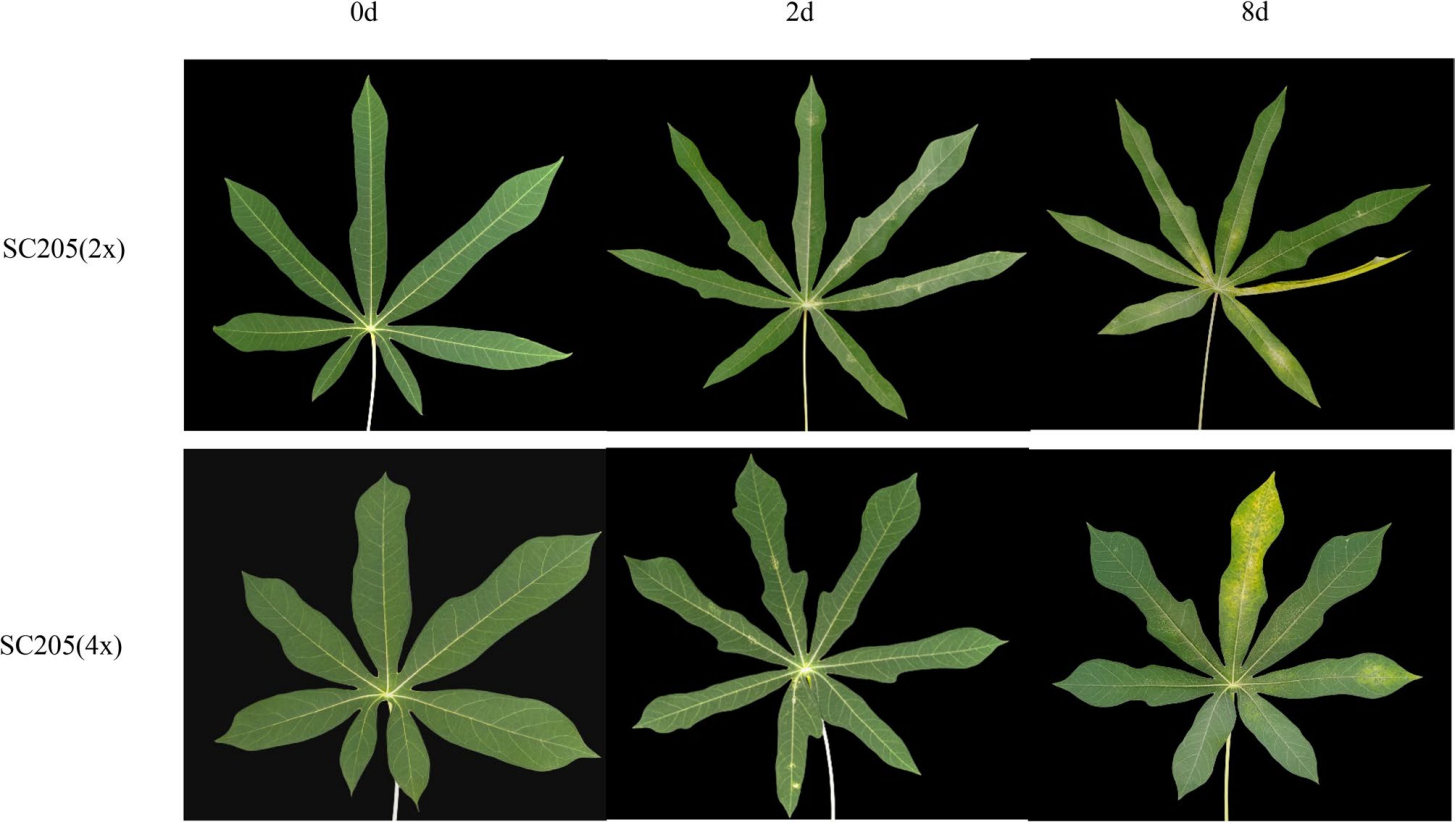
Morphological traits in diploid and autotetraploid cassava leaf under *T. cinnabarinus* stress

To verify the level of mite resistance, assessment of the degree of pest hazards was carried out after inoculated with leaves of SC205 (2×) and SC205 (4×) on 68 DAP. After 2 d of *T. cinnabarinus* feeding stress, small yellow white spots appeared on the surface of cassava leaves, and the spot areas on both SC205 (2×) (16.33 ± 2.85%, *n* = 45) and SC205 (4×) (8.87 ± 2.14%, *n* = 45) leaves were all less than 25% of the leaf area (Fig. 1), of which the mite damage level can be determined as level 1. For the 8 d treatment, the damage level increased on both genotypes. More yellow spots occurred on the surface of cassava leaves than the 2 d treatment. The damage spot area of SC205 (2×) leaves increased to 78.56% of the leaf area, with some cracks curling and withering, and the mite damage level reached to level 4. For SC205 (4×), the damage spot area of leaves increased to 51–75% (65.11 ± 4.79%, *n* = 45) of the leaf area counting to level 3 with no crack curling or drying (Fig. 1).

### Physiological response of the diploid and autotetraploid cassava under *T. cinnabarinus* stress

**Fig. 2:**
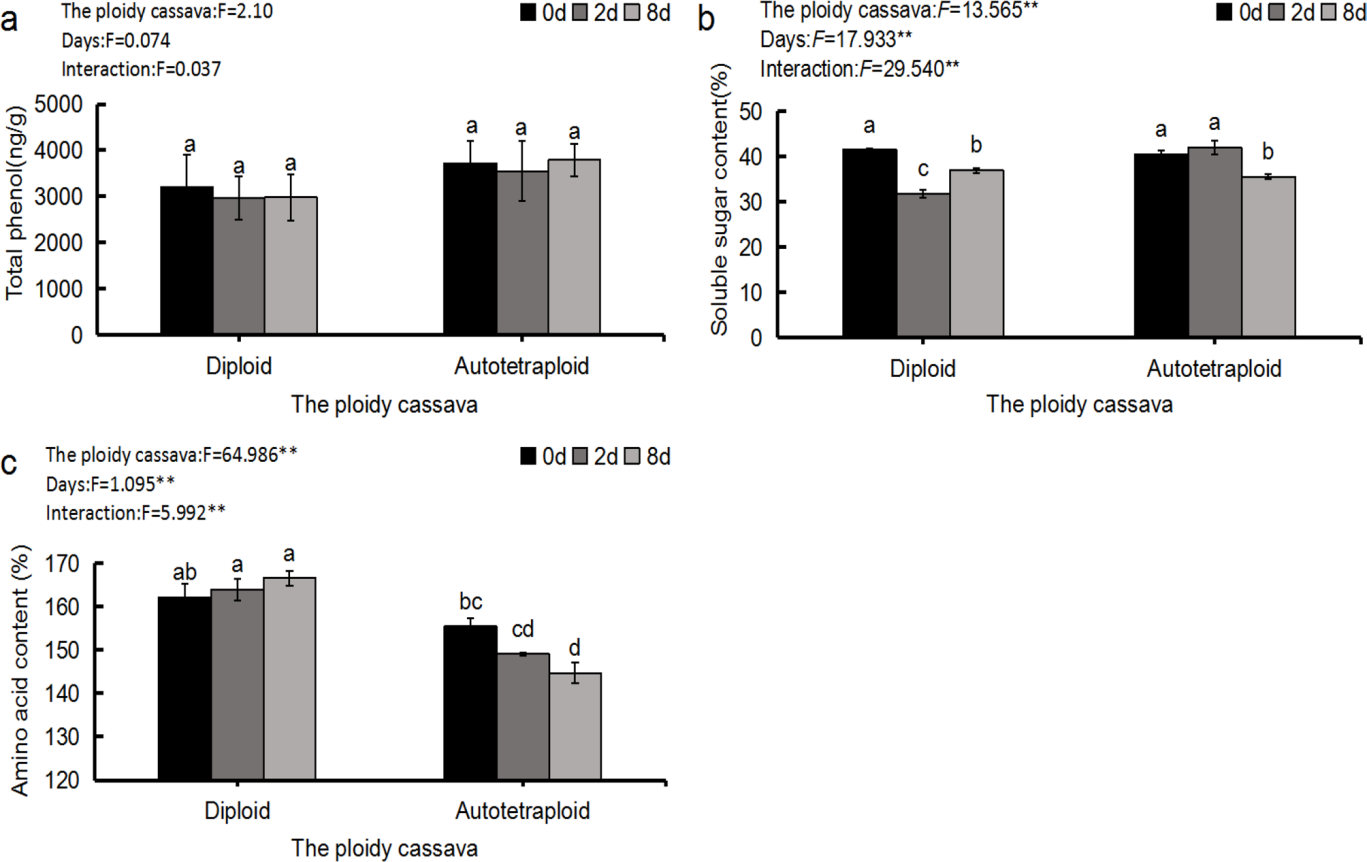
Total phenol (**a**), soluble sugars (**b**) and amino acids (**c**) under *T. cinnabarinus* stress. Note: *F*represent*F*-value of ANOVA; the same lower case letters in the figure indicate no significant difference and different letters indicate a significant difference (*P*<0.05); * indicates the significant difference at 0.05 level; * * indicates the significant difference at 0.01 level

ANOVA revealed that contents of soluble sugar and amino acids of cassava leaves under mites infection varied with genotype and infection time (Fig. 2b and c), while the contents of total phenol was quite stable among genotype and infection time (Fig. 2a). The contents of total phenol of SC205 (4×) were slightly higher but not significant than that of diploid cassava. Both the soluble sugar contents of SC205 (2×) under the mite infection for two days and eight days were all significantly lower than the 0 day infection treatment (Fig. 2b). In SC205 (4×), the soluble sugar content of cassava was slightly increased at the low-level foliar damage but significantly decreased when the damage level increased. The soluble sugar content of SC205 (2×) under the mite infection for two days was significantly lower than that of SC205 (4×) (Fig. 2b). The amino acid content of diploid cassava was increased gently with the degree of pest hazards, while a contrary trend was found in autotetraploid cassava (Fig. 2c).

### DEGs of different ploidy cassava under *T. cinnabarinus* stress

**Fig. 3:**
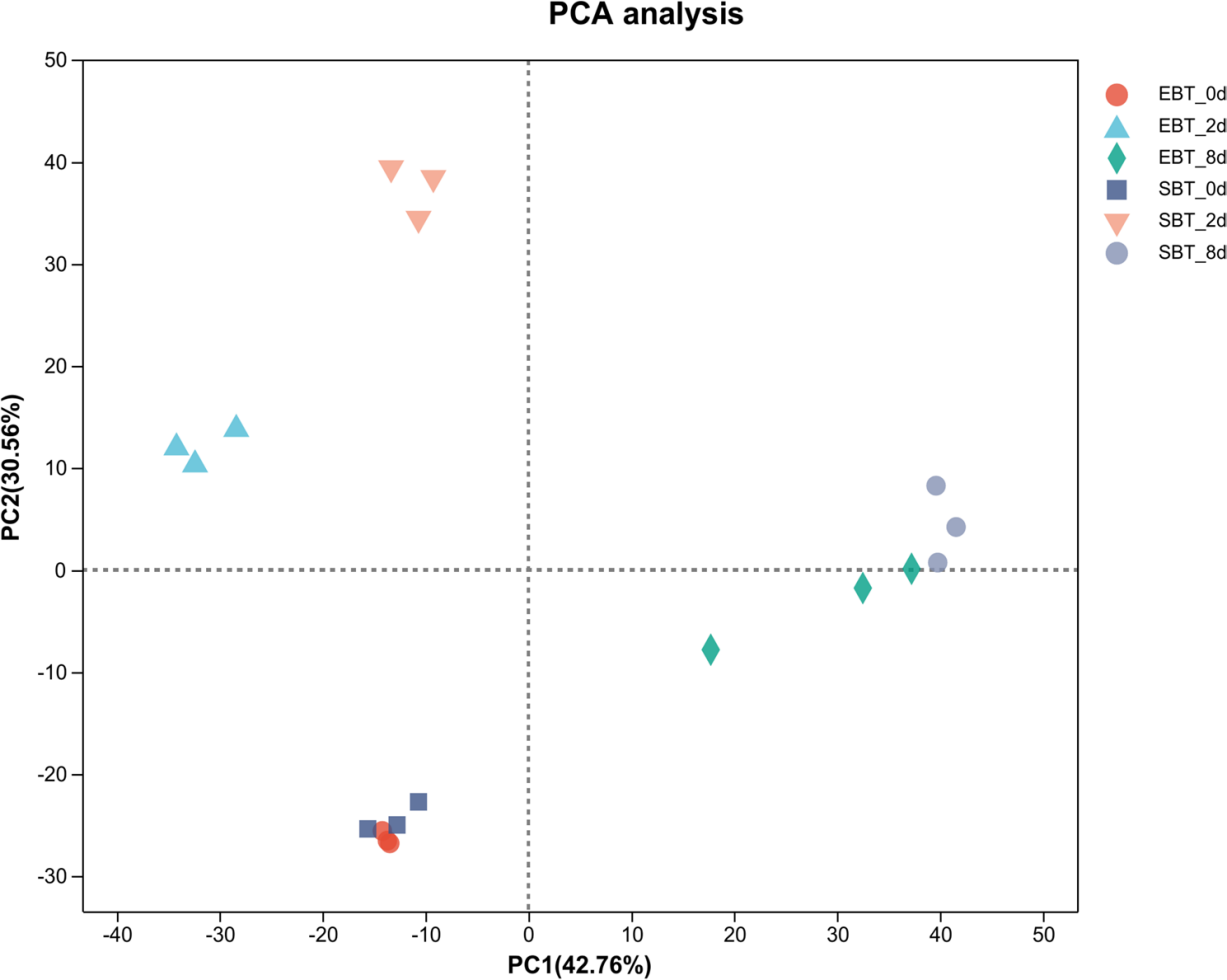
PCA scatter plot of gene expression variation across biological replicates, categorized by different ploidy levels and mite infection time points. Note: Samples EBT-0d, EBT-2d, and EBT-8d represent the SC205 (2×) treatment group at 0, 2, and 8 days mite infection, respectively. Samples SBT-0d, SBT-2d, and SBT-8d represent the SC205 (4×) treatment group at 0, 2, and 8 days mite infection, respectively

A total of 121.73 Gb of clean data were obtained, 6.06 Gb for each sample, and the Q30 base percentage was more than 94.02%, the results of sequence alignment were shown in Table A2. Principal component analysis was applied to overview the RNA-seq data (Fig. 3). Combing the correlation heatmap between samples, correlation between replicates is good, indicating the required data was reliable for further analysis (Fig. A1). To further validate RNA-seq data, eight DEGs between treatments were randomly chosen for qRT-PCR, and the results showed that the transcriptional levels of these genes were consistent with the transcriptome data, indicating the reliability of the RNA-seq data (Fig. A3). The samples were separated by mites infection days in the scatting plot by the first and the second components and biggest difference of the RNA-seq data between SC205 (2×) and SC205 (4×) can be found in the 2 d treatment among all treatments, which could suggest that cassava diploid and its autopolyploid may have different mechanisms or intensities of response to the mite feeding stress and potentially represent the peak of transcriptional activity induced by mite infestation and an ideal window for discovering stress-responsive genes (Fig. 3). The replicates for both non-infested SC205(2×) and SC205(4×) clustered closely together but were distinct from all other treatments. This indicates that their gene expression profiles were similar to each other but different from the mite-infected groups. The close clustering of SC205(2×) and SC205(4×) after 8 days infection suggests a similar transcriptional state. This, coupled with increased intra-group variability, may indicate that the plants are either recovering from or stabilizing after the applied stress.

**Fig. 4:**
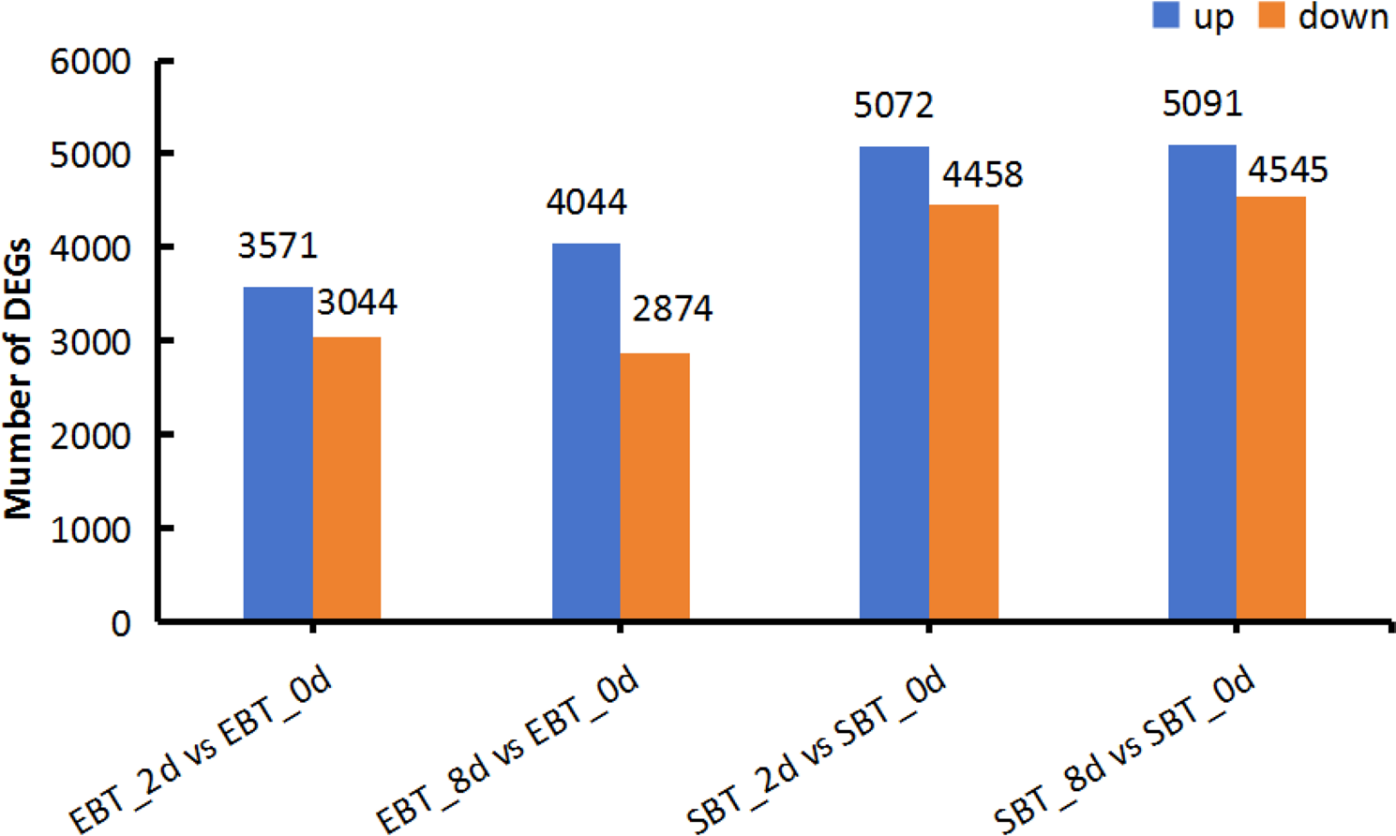
The numbers of DEGs in the distinct contrasting groups

The numbers of DEGs in diploid and autotetraploid cassava increased with the pest infection time (Fig. A2). Compare to the 0-day mite infection treatment, there were 6615 DEGs were found in the 2 d treatment of SC205 (2×) with 3571 up-regulated and 3044 down-regulated genes, while 6918 DEGs were found in 8 d treatment with 4044 up-regulated and 2874 down-regulated genes. For SC205 (4×), there were 9530 DEGs were found in the SBT_2d_vs_SBT_0d group with 5072 up-regulated and 4458 down-regulated genes, while 9636 DEGs were found in SBT_8d_vs_SBT_0d group with 5091 up-regulated and 4545 down-regulated genes (Fig. 4).

**Fig. 5:**
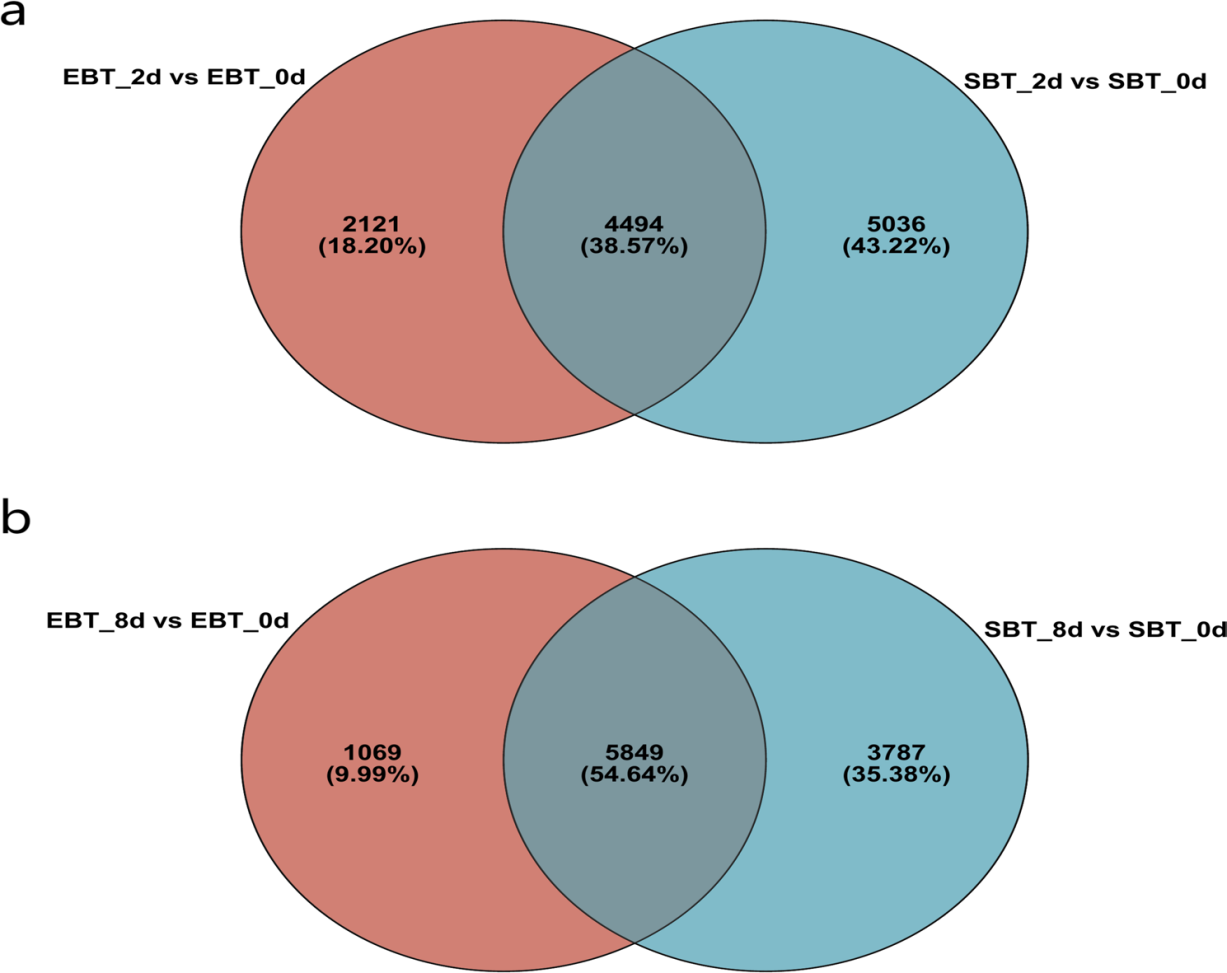
Venn analysis of DEGs in SC205 (2×)(EBT) and SC205 (4×)(SBT) under 2 d (**a**) and 8 d (**b**) pest hazards

**Fig. 6:**
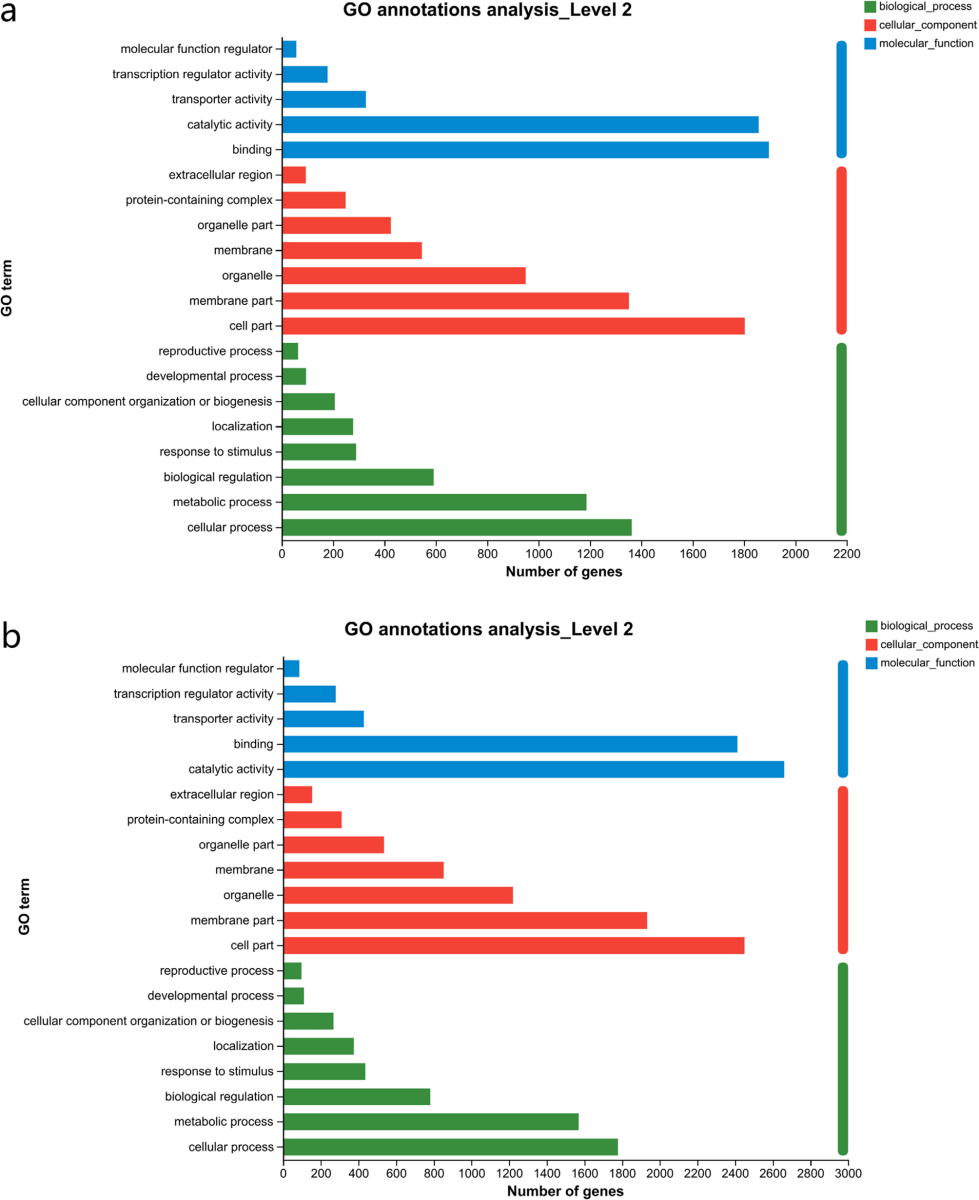
GO analysis of the DEGs shared between SC205 (2×) and SC205 (4×) under 2-day (4494 DEGs, **a**) and 8-day (5849 DEGs, **b**) pest hazards

Venn diagrams showed that 4494 DEGs were shared by SC205 (2×) and SC205 (4×) under 2 d pest hazards, while 2121 DEGs and 5036 DEGs were observed for SC205 (2×) and SC205 (4×), respectively (Fig. 5a). Under 8 d pest hazards, 5849 DEGs were shared by SC205 (2×) and SC205 (4×), while 1069 DEGs and 3787 DEGs were observed for SC205 (2×) and SC205 (4×), respectively (Fig. 5b). We annotated these shared DEGs of different ploidy levels, respectively. The annotation was performed against the top 20 terms from the GO database for each group (Fig. 6). These two shared DEG groups from both SC205 (2×) and SC205 (4×) under 2-day and 8-day pest infestation were annotated to 5 molecular function, 7 cellular component, and 8 biological process GO terms, respectively, while majority DEGs of them were all enriched in catalytic activity (GO:0003824), binding (GO:0005488), cell part (GO:0044464), membrane part (GO:0044425), cellular process (GO:0009987), and metabolic process (GO:0008152).

Furthermore, the results of GO annotation of the 2121 specific DEGs of SC205 (2×) under 2 d infection (Fig. A4a and A6a) showed similar trends as the shared DEGs (Fig. 6), including 5 items were classified as molecular function, 7 items as cellular component, and 8 items as biological process. For those 5036 specific DEGs of SC205 (4×) under 2 d infection (Fig. A4b and A6b), there were 6 items classified as molecular function, 7 items as cellular component, and 7 items as biological process. In the molecular function, molecular function regulator was unique to the specific DEGs of SC205 (4×) under 8 d pest hazards.

**Fig. 7:**
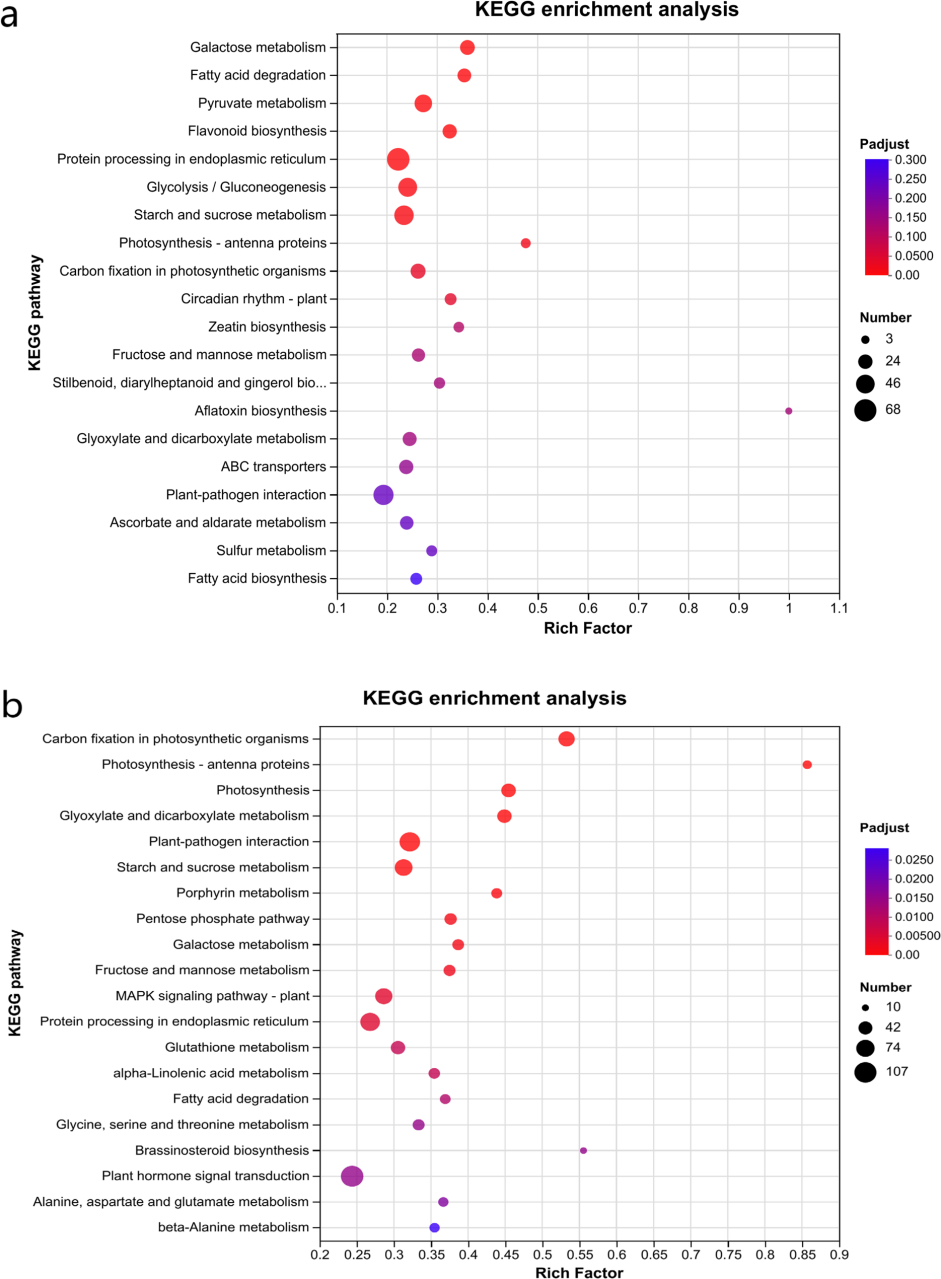
KEGG analysis of of DEGs shared between SC205 (2×) and SC205 (4×) under 2-day (4494DEGs, **a**) and 8-day (5849 DEGs, **b**) pest hazards

The KEGG analysis revealed that there were 10 significantly enriched KEGG pathways identified for both the SC205 (2×) and SC205 (4×) under 2 d pest hazards, such as galactose metabolism (map0052), fatty acid degradation (map00071), pyruvate metabolism (map00620), flavonoid biosynthesis (map00941) and so on (Fig. 7a, Table A3). The specific DEGs of SC205 (2×) were significantly enriched in flavone and flavonol biosynthesis (map00944) (*gene-LOC11062934*3, *gene-LOC110627778*, *gene-LOC110617654* and *gene-LOC110629198*) (Fig. A5a), while specific DEGs of SC205 (4×) were significantly enriched in Plant-pathogen interaction (map04626) (Fig. A5b). In addition, the shared DEGs, which were significantly enriched in KEGG pathways and regulated in opposite directions in SC205 (2×) compared to SC205 (4×) contrasts at each time points, were further investigated (Table A4). Following two days of mite herbivory, key genes in the starch and sucrose metabolism pathway (map00500; *LOC110606388*, *LOC110606650*, and *LOC110606650*) were up-regulated in SC205 (4×) but down-regulated in SC205 (2×), resulting in reduced soluble sugar content in the latter (Fig. 2b).

When cassava under 8 d *T. cinnabarinus* feeding stress, there were 23 significantly enriched KEGG pathways were identified for both the SC205 (2×) and SC205 (4×), which were plant-pathogen interaction (map04626), starch and sucrose metabolism (map00500), plant hormone signal transduction (map04075) and so on (Fig. 7b, Table A3). The specific DEGs of SC205 (2×) were mostly enriched in zeatin biosynthesis (map00908), plant hormone signal transduction (map04075) and amino sugar and nucleotide sugar metabolism (map00520) (Fig. A7a), while specific DEGs of SC205 (4×) were mostly enriched in pyruvate metabolism (map00620), fatty acid biosynthesis (map00061), endocytosis (map04144) and aflatoxin biosynthesis (map00254) (Fig. A7b). Furthermore, the chalcone synthase genes (*LOC110612286* and *LOC110612862*), which were found enriched in the flavonoid biosynthesis pathway, were all involved in the response to mite infection (Table A3). Gene *LOC11061228*6 was significantly upregulated following 2-day and 8-day infections in the SC205 (4×) group, and after the 2-day infection in the SC205 (2×) group. Gene *LOC110612862* was significantly upregulated following 2-day and 8-day infections in the SC205 (2×) group, and after the 8-day infection in the SC205 (4×) group.

### Metabolome analysis of different ploidy cassava under *T. cinnabarinus* stress

**Fig. 8:**
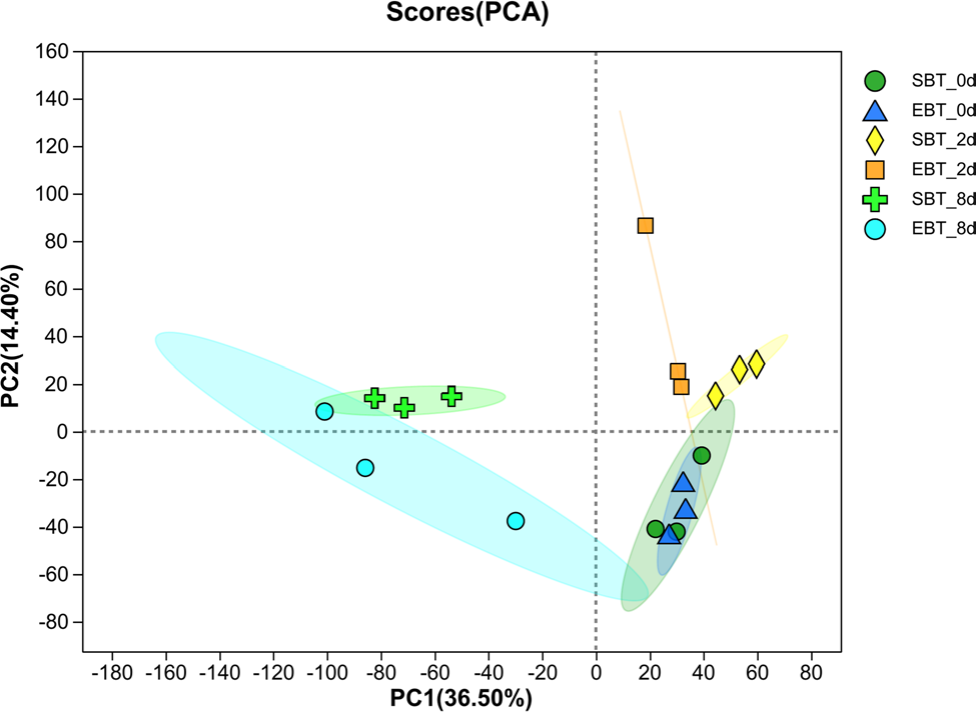
PCA scatter plot of metabolites variation across biological replicates, categorized by different ploidy levels and mite infection time points. Note: Samples EBT-0d, EBT-2d, and EBT-8d represent the SC205 (2×) treatment group at 0, 2, and 8 days mite infection, respectively. Samples SBT-0d, SBT-2d, and SBT-8d represent the SC205 (4×) treatment group at 0, 2, and 8 days mite infection, respectively

A total of 1758 metabolites were identified among all samples, including 988 metabolites in negative ion mode and 770 metabolites in positive ion mode. Principal component analysis showed that the first and the second components explained 50.9% of the variation and the cassava samples were mainly varied with the pest hazards time (Fig. 8). The correlation heatmap between samples also showed that correlation between replicates is good and samples under 8 d pest hazards were negatively correlated with samples under 0 d and 2 d pest hazards, indicating the required data was reliable for further analysis (Fig. A8).

**Fig. 9:**
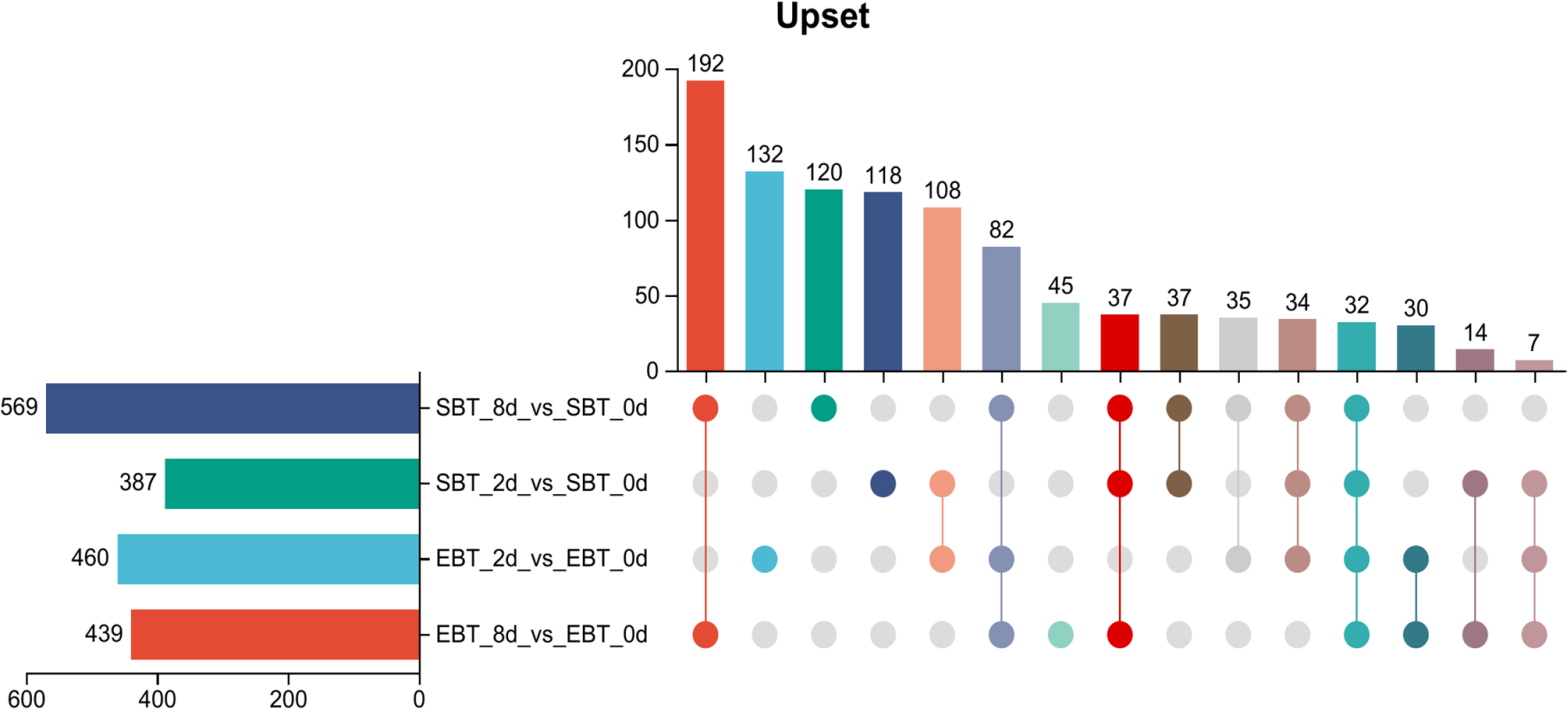
Upset analysis of differentially accumulated metabolites in the distinct contrasting groups

By combining OPLS-DA modelling and *t*-test analysis, metabolites between different groups were screened as differentially accumulated metabolites (DAMs) based on the criteria of VIP > 1, *P* < 0.05, and |FC|=>2. The OPLS-DA models showed that samples under the 2 d and 8 d pest hazards were all showing a clear separation from the 0 d pest hazards group, indicating that significant changes occurred in the metabolites of diploid and autotetraploid cassava under *T. cinnabarinus* infection (Fig. A9). In the diploid cassava, there were 460 DAMs found in the 2 d vs. 0 d pest hazards group with 249 DAMs up-regulated and 211 DAMs down-regulated, while there were 439 DAMs found in the 8 d vs. 0 d pest hazards group with 307 DAMs up-regulated and 132 DAMs down-regulated. In the autotetraploid cassava, there were 387 DAMs in the 2 d vs. 0 d pest hazards group with 81 DAMs up-regulated and 306 DAMs down-regulated, while there were 569 DAMs in the 8 d vs. 0 d pest hazards group with 360 DAMs up-regulated and 209 DAMs down-regulated (Fig. A10). The numbers of DAMs in autotetraploid cassava increased with the pest infection time, while DAMs of diploid cassava showed opposite trends (Fig. 9).

**Fig. 10:**
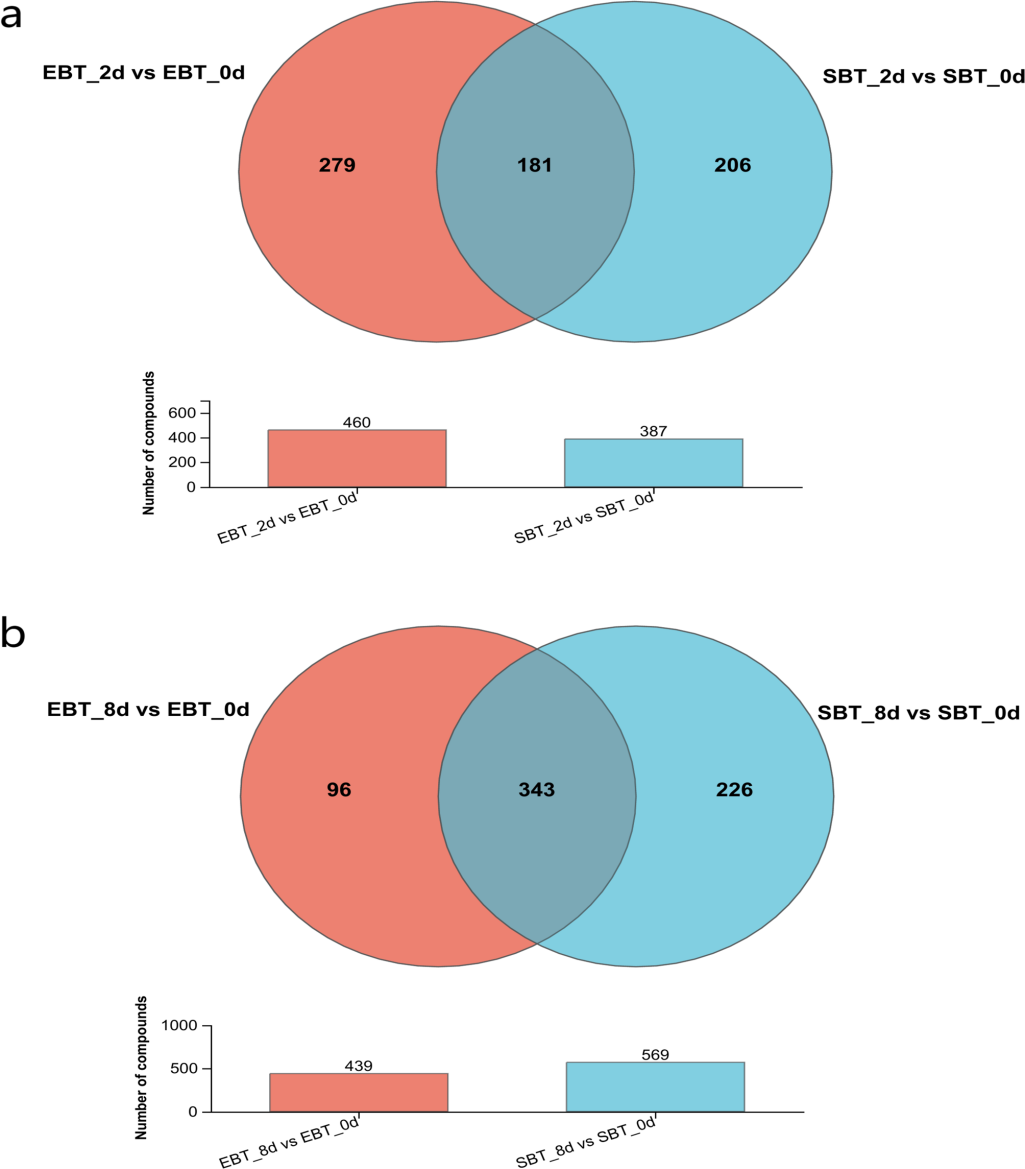
Venn analysis of DAMs in SC205 (2×)(EBT) and SC205 (4×)(SBT) under 2 d (**a**) and 8 d (**b**) pest hazards

There were 181 DAMs shared by SC205 (2×) and SC205 (4×) under 2 d pest hazards, while 279 DAMs and 206 DAMs were observed for SC205 (2×) and SC205 (4×), respectively (Fig. 10a). Under 8 d pest hazards, 343 DAMs were shared by both the SC205 (2×) and SC205 (4×), while 96 DAMs and 226 DAMs were observed for SC205 (2×) and SC205 (4×), respectively (Fig. 10b).

**Fig. 11:**
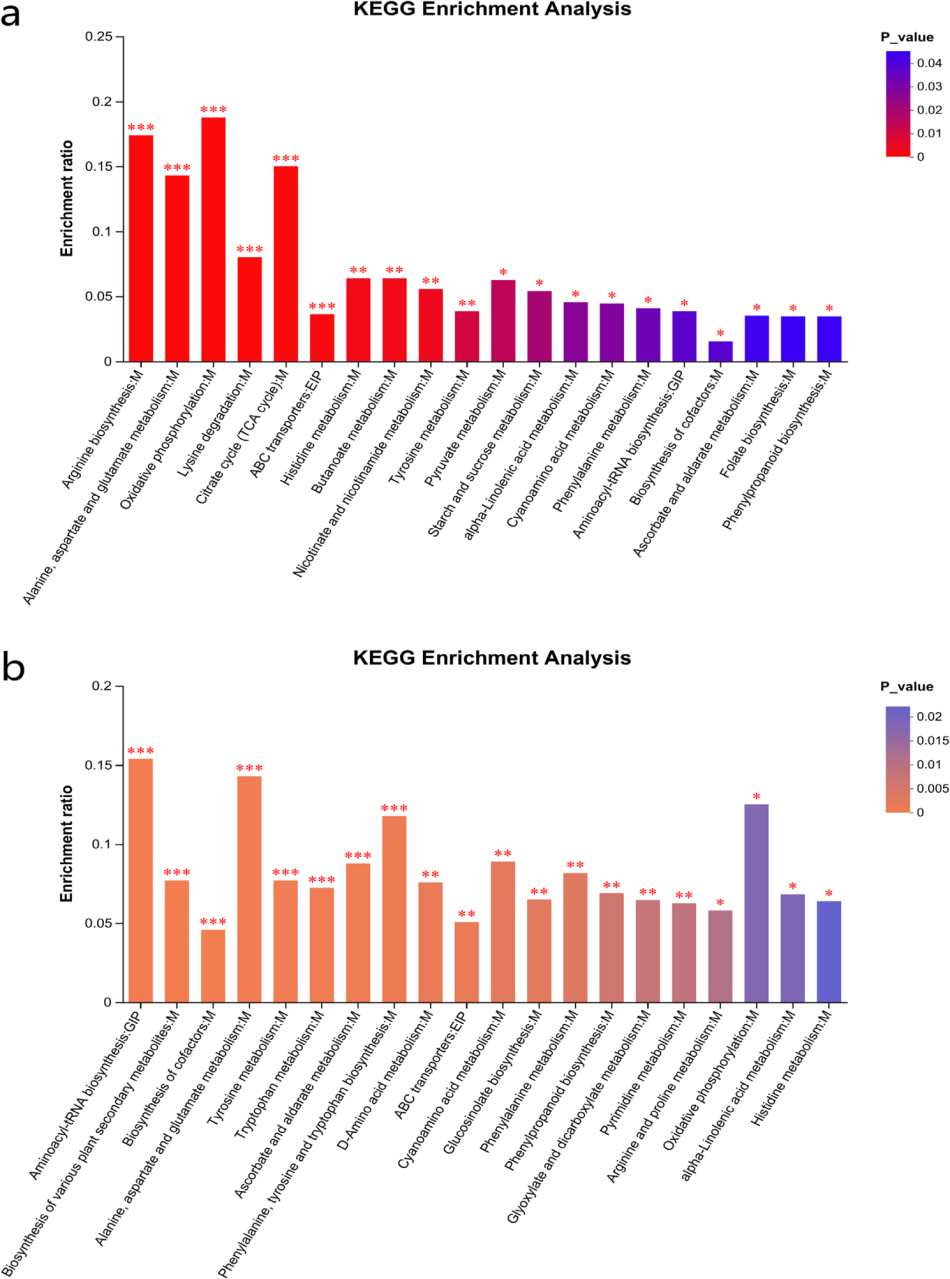
KEGG analysis in 181 DAMs of cassava under two-day mite infection (**a**) and 343 DAMs of cassava under eight-day infection (**b**). Note:* indicates the significant difference at 0.05 level,* * indicates the significant difference at 0.01 level,*** indicates the significant difference at less than 0.01 level

Furthermore, the KEGG enrichment analysis revealed that 181 shared DAMs of cassava under the 2 d pest hazards were significantly enriched in energy metabolism (map00190), carbohydrate metabolism (map00020), amino acid metabolism (map00350), and other multiple channels (Fig. 11). The 279 unique DAMs of SC205 (2×) were significantly enriched in 5 pathways, which were amino acid metabolism (map00400 and map00350, mainly in the aspect of phenylalanine, tyrosine and tryptophan biosynthesis and tyrosine metabolism), lipid metabolism (map00592, mainly in the aspect of alpha-Linolenic acid metabolism), and biosynthesis of other secondary metabolites (map00999 and map00944, mainly in the aspect of biosynthesis of various plant secondary metabolites and flavone and flavonol biosynthesis) (Fig. A11a). A total of 27 metabolites were significantly enriched in these five KEGG pathways, including 10 up-regulated metabolites and 17 down-regulated metabolites. Most of these ten up-regulated metabolites were mainly concentrated in alpha-linolenic acid metabolism and biosynthesis of various plant secondary metabolites. In the meanwhile, the unique DAMs of SC205 (4×) were also significantly enriched in 5 pathways, which were amino acid metabolism (map00250, map00220 and map00330, mainly in the aspect of alanine, aspartate and glutamate metabolism, arginine biosynthesis and arginine and proline metabolism), lipid metabolism (map00591, mainly in the aspect of linoleic acid metabolism), and membrane transport (map02010, mainly in the aspect of ABC transporters) (Fig. A11b). A total of 17 metabolites were significantly enriched in these five KEGG pathways, including one up-regulated metabolite (trehalose) and 16 down-regulated metabolites.

Within the 8 d *T. cinnabarinus* infection group, the 343 shared DAMs were significantly enriched in aminoacyl-tRNA biosynthesis (map00970), biosynthesis of various plant secondary metabolites (map00999), biosynthesis of cofactors (map01240), and other multiple channels showed in Fig. 11b. The 96 unique DAMs of SC205 (2×) were significantly enriched in two pathways, carbohydrate metabolism (map00052, galactose metabolism) and amino acid metabolism (map00290, valine, leucine and isoleucine biosynthesis) (Fig. A12a). A total of 5 metabolites were significantly enriched in these two KEGG pathways, while the up-regulated metabolites were stachyose, galactosylglycerol and L-threonine and the down-regulated metabolites were D-galactose and (S)−2-aceto-2-hydroxybutanoic acid. The 226 unique DAMs of SC205 (4×) were significantly enriched in 3 pathways, biosynthesis of other secondary metabolites (map00940, phenylpropanoid biosynthesis), carbohydrate metabolism (map00052, galactose metabolism) and amino acid metabolism (map00310, lysine degradation) (Fig. A12b). A total of 16 metabolites were significantly enriched in these three KEGG pathways, including 8 up-regulated metabolites and 8 down-regulated metabolites.

### Integrative analysis of transcriptome and metabolome

**Fig. 12:**
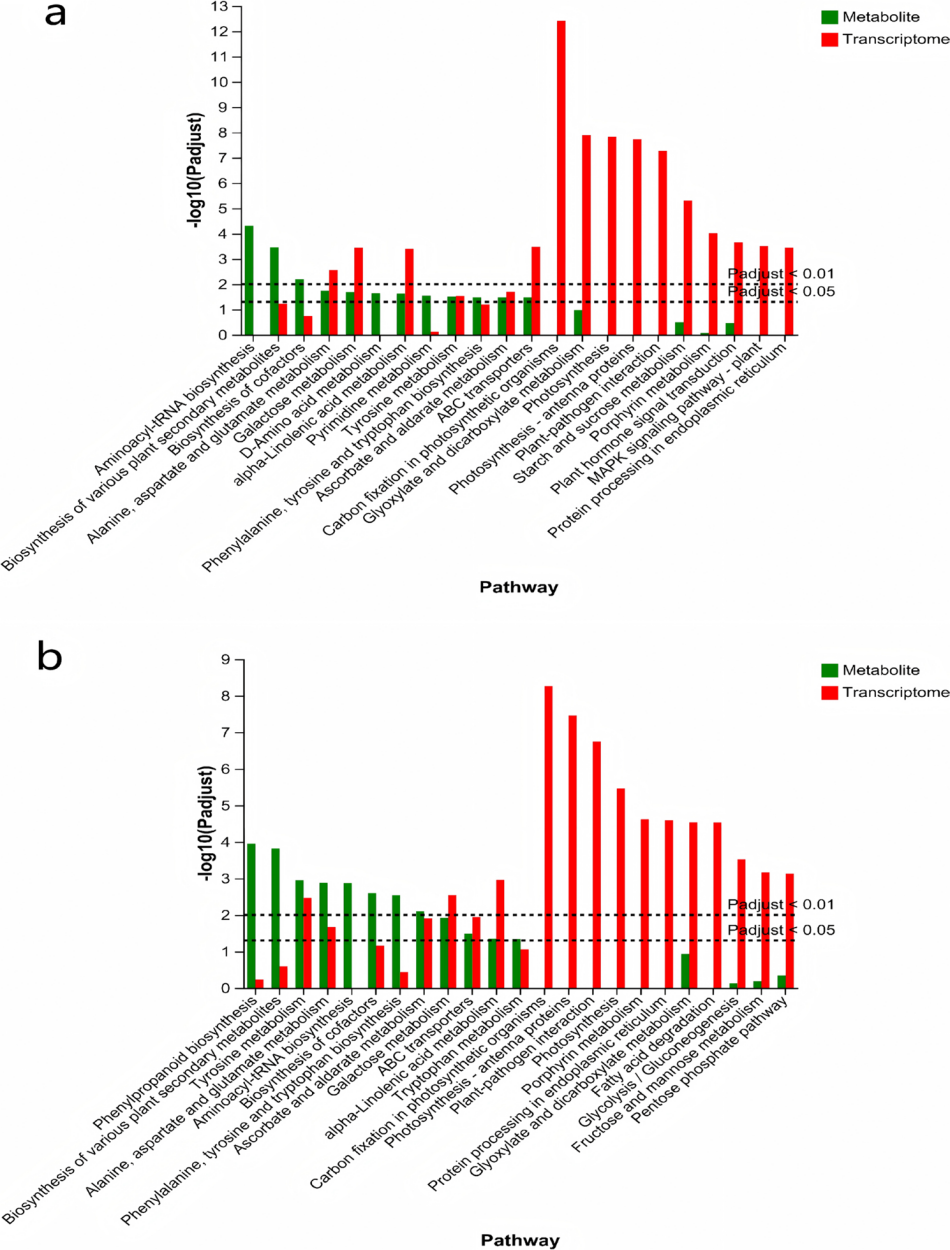
KEGG analysis of the metabolome and transcriptome in the 8 d pest hazards groups, categorized by different ploidy levels (EBT_8d vs. EBT_0d, **a**; SBT_8d vs. SBT_0d, **b**)

In order to better understand the relationship between DEGs and DAMs, a joint analysis was conducted. The results revealed that DEGs and DAMs in the each group were found co-enriched in a few KEGG pathways. In 2 d *T. cinnabarinus* feeding groups, both the DEGs and DAMs of SC205 (2×) were found significantly co-enriched in biosynthesis of other secondary metabolites (mesc00944, flavone and flavonol biosynthesis) (Fig. A13a), while DEGs and DAMs of SC205 (4×) were found significantly co-enriched in amino acid metabolism (mesc00330, arginine and proline metabolism) (Fig. A13b). In 8 d *T. cinnabarinus* feeding groups, the DEGs and DAMs of SC205 (2×) and SC205 (4×) were all found significantly co-enriched in six identical KEGG pathways, which were lipid metabolism (mesc00592, alpha-linolenic acid metabolism), membrane transport (mesc02010, ABC transporters), carbohydrate metabolism (mesc00052 and mesc00053, galactose metabolism and ascorbate and aldarate metabolism), and amino acid metabolism (mesc00250 and mesc00350, alanine, aspartate and glutamate metabolism and tyrosine metabolism) (Fig. 12).

## Discussion

Cassava is a globally important food and cash crop, providing carbohydrates source for nearly 1 billion people in more than 100 countries [33, 34]. However, the occurrence of various diseases and insect pests in cassava agricultural production significantly limits its yield and quality, including *T. cinnabarinus*, *Green Mite*, *Bacterial Blight*, and *Mosaic Disease* [35–38]. In particular, the damage caused by *T. cinnabarinus* on cassava become more and more serious in China, leading to significant cassava production loss [6, 9]. Therefore, it is essential to prevent and control the occurrence of *T. cinnabarinus* in agricultural production. In cassava, structural traits such as leaf cell wall thickness and fence tissue thickness form the first physical barrier to feeding by the mite. Some studies have found that the leaf thickness, middle fissure leaf width, fence tissue thickness and sponge tissue thickness of cassava autotetraploid are all higher than that of cassava diploid [27]– [28]. In the current study, SC205 (4×) were less affected and displayed significantly higher resistance to *T. cinnabarinu*s infection than SC205 (2×) (Fig. 1), which is consistent with previous studies [27, 28].

When the pest colonizes the plant, the nutrients (such as soluble sugars, soluble proteins, amino acids, etc.) or toxic secondary metabolites (phenols, alkaloids, etc.) release in the plant, which can directly affect the growth and the development of the pest and then increase the plant insect resistance. Phenolic compounds was proved to can affect the cell membrane of insect intestinal epidermis, making insect diet, which is not conducive to insect growth and development [39]. Various secondary metabolites, including phenylpropanoids, flavonoids, and terpenoids, confer resistance to herbivory or function as communication signals between plants and insects [40]. Secondary metabolites can help plants interact with their environment, especially in adverse or stressful conditions, which play an important role in the development and growth of plant species by providing defense against pests [41]. Under heat and drought stress, soluble sugar and amino acid contents were found increased in wheat, resulting in increased aphid resistance [42]. In the current study, although the difference was not statistically significant, the total phenolic contents of SC205 (4×) among all treatments were slightly higher than those of SC205 (2×), while its amino acids were decreasing and consistently lower than SC205 (2×) (Fig. 2), creating an environment that was not conducive to the growth, development and reproduction of *T. cinnabarinus*.

Integrated transcriptomics and metabolomics analyses is now commonly used to assess plant-pest interactions, including systemic acquired resistance and induced resistance [43–45]. In this study, the *T. cinnabarinus* infestation triggered the expression of many genes and various metabolic processes reaction. The DEGs and DAMs variation trend is consistent, suggesting a strong correlation (Fig. 12). Most DAMs of SC205 (2×) and SC205 (4×) were enriched in carbohydrate metabolism and amino acid metabolism under the 2 d and 8 d pest hazards, and integrative analysis of DEGs and DAMs also significantly enriched in these two pathways. Amino acid metabolism and carbohydrate metabolism are crucial to the salt stress response, which are responsible for biological cell adaptation, and the main players in various metabolic and regulatory pathways [46]. Interestingly, there were more DEGs observed in SC205 (4×) than in SC205 (2×) after 2 days or 8 days *T. cinnabarinus* infestation (Fig. 4). The DAMs identified in SC205 (2×) under 2 days of pest infestation were significantly enriched in biosynthesis pathways of various plant secondary metabolites. However, SC205 (4×) is mainly preliminary against the mechanical damage under 2 days pest hazards because of its thick leaves (Wei et al., 2018) and its DAMs significantly enriched in ABC transporters, while the DAMs were found significantly enriched in biosynthesis of various plant secondary metabolites under the 8 d pest hazards, producing secondary metabolites to fight the mite.

**Fig. 13:**
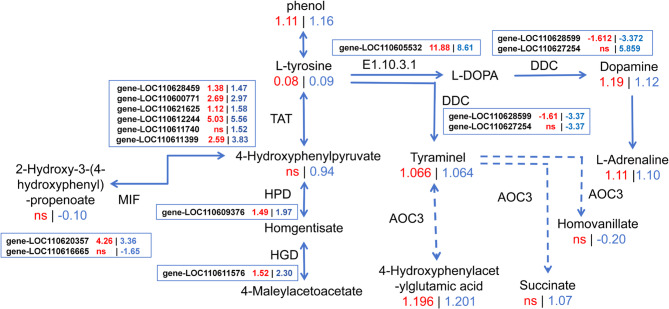
Network of the tyrosine metabolism pathway in the 8 d pest hazards. The location of the node is metabolites and the substances on the line are enzymes. Some of them that were unaffected by pest hazards have been omitted from this graph. The red numbers represent the |log2fold change| of enzyme or metabolites for SC205 (2×), while the blue number represents the |log2fold change| of enzyme or metabolites for for SC205 (4×). Note：ns，no significant

As the basic unit of protein synthesis, amino acids are able to act as osmoregulatory substances and maintain biofilm stable during plant stress [47]. Several amino acids serve as precursors for the synthesis of secondary metabolites [48]. Many studies also found that amino acid metabolism is closely related to stress tolerance of plant [49–51]. There is study show that increases in aromatic amino acids (tyrosine) might enhance the accumulation of secondary metabolites [52]. In this study, the DEGs and DAMs of SC205 (2×) and SC205 (4×) were significantly co-enriched in tyrosine metabolism. There were 10 metabolites in SC205 (4×) and 6 metabolites in SC205 (2×) enriched in the tyrosine metabolism, respectively (Fig. 13). The secondary metabolism produced by tyrosine metabolism plays an important role in plant growth and development and resistance to environmental stresses [53]. And most secondary metabolites (4-hydroxyphenylacetylglutamic acid, succinate, homovanillate) were significantly enriched and significantly upregulated in SC205 (4×). In addition, 4-hydroxyphenylacetylglutamic is a precursor for biosynthesis of various secondary metabolites and compounds including plastoquinone, tocopherols, rosmarinic acid and benzylisoquinoline alkaloids [54–57]. And tyrosine aminotransferase (TAT, EC 2.6.1.5) is a key enzyme in the tyrosine-derived pathway in rosmarinic acid biosynthesis [58]. TAT enzymes catalyze the reversible interconversion of tyrosine and 4-hydroxyphenylpyruvate, which is the first step in the tyrosine-derived pathway [54–59]. Some studies have found that overexpression of a TAT gene increased the rosmarinic acid yield, which is capable of scavenging free radicals and enhance protection against abiotic and biotic stresses [55, 60]. In our study, ANOVA based on a generalized linear model was conducted using the FPKM value of the expression of the enriched genes (*gene-LOC110628459*, *gene-LOC110600771*, *gene-LOC110621625*, *gene-LOC110612244*, *gene-LOC110611740*, and *gene-LOC110611399*), incorporating both ploidy level and time as the main factors along with their interaction. Although the difference was not statistically significant, the expression of these genes were found higher in the TAT of SC205 (4×) than that of SC205 (2×), ending up more tyrosine and their derivatives than the latter (Fig. 13) and stronger demonstrated a stronger ability to resist mites (Fig. 1). Furthermore, the alanine, aspartate and glutamate metabolism was also found as important pathway for cassava in response to *T. cinnabarinus* stress, which were closely related to plant tolerance/detoxification mechanisms [61]. There were 6 DAMs and 10 DEGs enriched in this pathway, most of them were involved in the Citrate cycle and amino acid synthesis. Aspartate serves as a precursor for the biosynthesis of a variety of amino acids and their derived metabolites, which play essential roles in plant growth, reproduction, development, and defense [62]. In this study, the aspartate of SC205 (2×) and SC205 (4×) were all up-regulation, leading to an increase in L-glutamine and L-1-pynoline-5-canboxylate (Fig. A14a).

In the carbohydrate metabolism pathway, ascorbic acid (AA, vitamin C) is a multifaceted molecule with diverse physiological functions in plants [63], and particularly well-known for its roles in photosynthetic functions and stress tolerance. It is characterized as an antioxidant that counteract oxidative stress produced by normal or stressed cellular metabolism, and particularly as a ROS scavenger [64–66]. It is known that AA is synthesized of L-galactose pathway, the myoinositol pathway and so on [67–69]. An explanation for the elevated AA levels could be an expression of plant stress resistance [70]. However, it has been suggested that under the pathogen attack, a decrease in ascorbate could lead to an oxidative burst which might prevent bacterial infection [71, 72]. In this study, the L-galactose of SC205 (2×) and SC205 (4×) were all down-regulation (Fig. A14b). However, the AA was only down-regulation in the SC205 (4×). Thus, we conferred that the reduction of AA can increase the mite resistance of SC205 (4×). It was demonstrated recently that Vitamin C deficient 4 (VTC4, EC 2.7.4.1) is a bifunctional enzyme that not only affects AA but also myoinositol biosynthesis [73]. The *vtc* mutants also exhibit an array of pleiotropic developmental and metabolic defects, including increased resistance to virulent pathogens [74, 75], elevated levels of abscisic acid [76, 77], etc. In Arabidopsis, ascorbate-deficient *vtc* mutants, the reduction in the ascorbate level leads to enhanced resistance to virulent biotrophic pathogens [78]. Our results are consistent with previous studies and showing that the VTC4 genes (*gene-LOC110607532*, *gene-LOC122722194*, and *gene-LOC122724347*) were all down-regulated expression leading to the reduction of L-galactose and ascorbic acid.

Furthermore, membrane lipids and their derivatives play pivotal roles in inter- and intracellular signaling, and ultimately mediate organisms’ interactions with their stress environment. Besides their contribution as structural constituents of cellular membranes, lipids also serve as precursors for signaling metabolites that regulate plant growth, development and response to the environment [79, 80]. Jasmonic acid (JA), derived from α-linolenic acid via one branch of the octadecanoid pathway, is a key mediator of defense responses against chewing insects [81]. The α-Linolenic acid metabolism is the starting point of JA synthesis. When plants are damaged by pests or diseases, JA content increase rapidly, which can induce the activation of defense genes and the synthesis of secondary compounds, as well as improve the stress resistance of plants [82]. In this study, the JA relative content were found increased in two varieties and it was higher in SC205 (4×) than that of SC205 (2×), which is consistent with Zhou et al. [83]. And the related genes of JA biosynthesis (AOC, OPR, OPCL1, ACX, MFP2, and fadA) were all up-regulated expression in the SC205 (4×) and SC205 (2×), especially in the SC205 (4×)(Fig. A14c). All these results indicated that under the mite infestation, cassava synthesizes JA to initiate defense signaling pathways, thereby enhancing direct and indirect resistance to mites.

## Conclusion

In conclusion, cassava diploids and their autotetraploids were characterized for indoor resistance and their induced defense responses following *T. cinnabarinus* infestation were investigated using transcriptomic and metabolomic analyses. Leaf damage identification, nutrient and secondary metabolite results proved that SC205 (4×) was more resistant to *T. cinnabarinus* infestation than SC205 (2×). Comprehensive transcriptomics and metabolomics analyses indicated that amino acid and carbohydrate metabolism may play important roles in conferring resistance to the vermilion leaf mite in cassava diploids and their autotetraploids. However, the molecular mechanisms of mite resistance in cassava autotetraploids need further investigation. Overall, this study provides new insights into the understanding of the molecular and biochemical mechanisms of cassava resistance to *T. cinnabarinus*; furthermore, these findings could help in the identification of resistance genes and the breeding of resistant cassava varieties.

## Supplementary Information

Supplementary Material 1.

Supplementary Material 2.

## Data Availability

The transcriptome sequencing data were deposited in the NCBI SRA database under project number PRJNA1088828 (https://www.ncbi.nlm.nih.gov/sra/PRJNA1088828). The Metabolome data reported in this paper have been deposited in the BioProject in National Genomics Data Center, Beijing Institute of Genomics, Chinese Academy of Sciences/China National Center for Bioinformation, under accession number PRJCA041591 that is publicly accessible at https://ngdc.cncb.ac.cn/bioproject. The original data was included in the article and/or supplementary material. Further information can be accessed by contacting the corresponding author.

## Abbreviations

*T. cinnabarinus*: *Tetranychus cinnabarinus*
GSATAS: Guangxi South Asian Tropical Agricultural Science Research Institute
DEGs: Differentially expressed genes
DAMs: Differentially accumulated metabolites
GO: Gene Ontolog
KEGG: Kyoto Encyclopedia of Genes and Genomes
