# Supplementary material for: Multiomics analysis of cassava with different ploidy levels in response toTetranychus cinnabarinus
Source: BMC Plant Biol. 2025 Nov 7;25:1526. doi: 10.1186/s12870-025-07522-6 (PMC12595817; doi:10.1186/s12870-025-07522-6)
Supplement: Supplementary file 1 — Supplementary Material 1. [file 12870_2025_7522_MOESM1_ESM.docx]

Multiomics analysis of cassava with different ploidy levels

in responce to *Tetranychus cinnabarinus*

Wanling Wei ^1†^, Yuanhang Huang ^2†^ , Zhenling Huang ^1^, Haixia Yang ^1^, Zhaoqin Cai ^1^, Ruolan Huang ^1^, Wen He ^1^, Huixian Chen ^1^, Zhenhua Liang ^1^, Lixia Ruan ^1^, Xiu Lan ^1^, Qingwen Deng ^3^, Guanyong He ^1^, Qing Chen ^4^, Jinren Luo^5^, Maogui Wei ^3*^ and Hengrui Li ^1*^

**Table A1** Gene name and primer sequences

| **Gene name** | **Name of the primer** | **Primer sequences（5'to3'）** | **Product size(bp)** |
| --- | --- | --- | --- |
| Actin | Actin F | TGATGAGTCTGGTCCATCCA | 163 |
|  | Actin R | CCTCCTACGACCCAATCTCA |  |
| *LOC110602651* | LOC110602651 F | TCGATGCCAGCCTCAACAG | 174 |
|  | LOC110602651 R | CACTCTCCAACCTAGCCTTCAT |  |
| *LOC110616948* | LOC110616948 F | AAGAAGGAGCGGAGGAATACC | 168 |
|  | LOC110616948 R | CAGAATCAGGCAACTCAGAACA |  |
| *LOC110613700* | LOC110613700 F | TGGTATTGTCTGCTCGGTGTT | 174 |
|  | LOC110613700 R | GGTGGAGGAATCACATCTCTGA |  |
| *LOC110604189* | LOC110604189 F | CCTCCTCGGCGTCCTGTATA | 178 |
|  | LOC110604189 R | TTGCTGCTTATCAACCTGTGGA |  |
| *LOC110604210* | LOC110604210 F | ACACCAGAGTTGCGTCAGG | 104 |
|  | LOC110604210 R | ACATTAGTCCGAGCAGTAGCC |  |
| *LOC110614708* | LOC110614708 F | GTCCACTTGCCACATCTTAACTT | 113 |
|  | LOC110614708 R | CTCTGCTGCTCCTCACCTTG |  |
| *LOC110620242* | LOC110620242 F | TGGTGGCAATCAGATAGTAGCA | 105 |
|  | LOC110620242 R | GATGGCGGTGAAGACAGGAA |  |
| *LOC110616945* | LOC110616945 F | ATCCTCTGCTCAACACTCCTT | 157 |
|  | LOC110616945 R | AAGTTCCAGTCATCCGAGATTG |  |

**Table A2** The results of sample sequence alignment

| **Sample** | **Total reads** | **Total mapped** |
| --- | --- | --- |
| S401 | 43032820 | 41349923(96.09%) |
| S402 | 49431320 | 46874337(94.83%) |
| S403 | 40561798 | 38246463(94.29%) |
| S201 | 44242608 | 41874753(94.65%) |
| S202 | 46677888 | 44478174(95.29%) |
| S203 | 46581152 | 44333028(95.17%) |
| S421 | 44166676 | 41006854(92.85%) |
| S422 | 42679348 | 39505963(92.56%) |
| S423 | 43412556 | 40643796(93.62%) |
| S221 | 47454258 | 42927863(90.46%) |
| S222 | 45672514 | 41733421(91.38%) |
| S223 | 42230352 | 36798501(87.14%) |
| S481 | 44642746 | 42309057(94.77%) |
| S482 | 52014044 | 49595461(95.35%) |
| S483 | 46974454 | 44775293(95.32%) |
| S281 | 48464194 | 45992172(94.9%) |
| S282 | 43578726 | 41603283(95.47%) |
| S283 | 43327412 | 41211187(95.12%) |

Note: Samples EBT-0d (S201-S203), EBT-2d (S221-S223), and EBT-8d (S281-S283) represent the SC205 (2×) treatment group at 0, 2, and 8 days post-mite infection, respectively. Samples SBT-0d (S401-S403), SBT-2d (S421-S423), and SBT-8d (S481-S483) represent the SC205 (4×) treatment group at 0, 2, and 8 days post-mite infection, respectively.


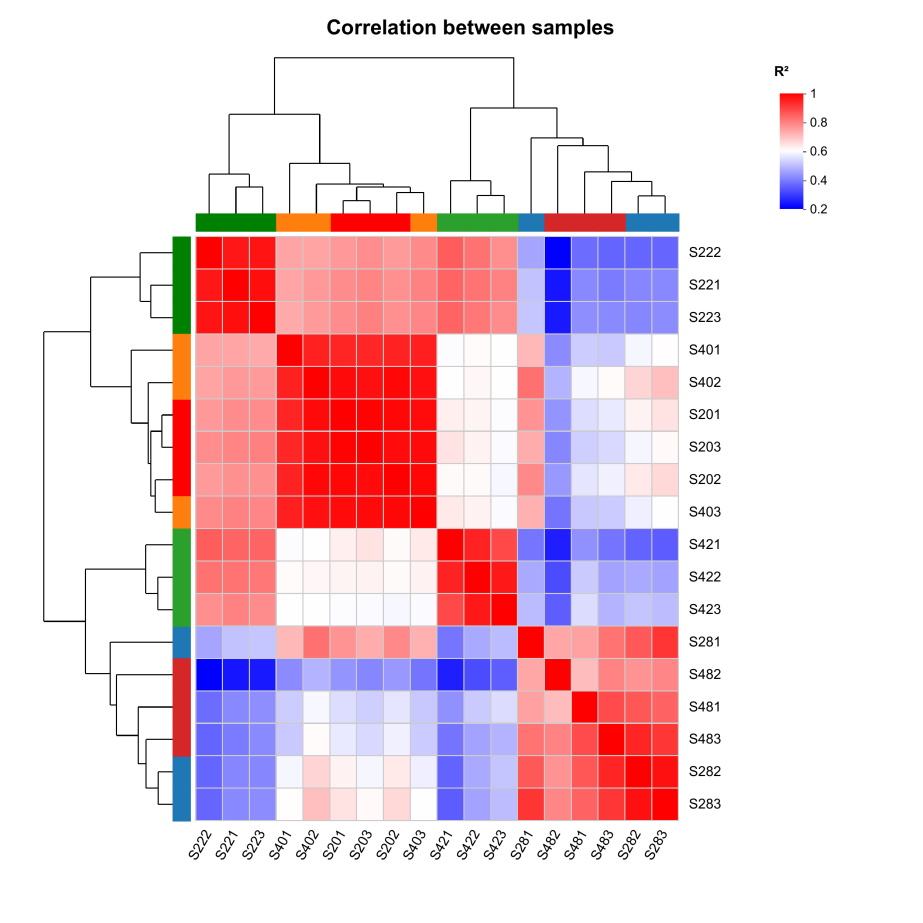
**Figure A1.** Correlation between samples in the transcriptome analysis.

Note: Samples EBT-0d (S201-S203), EBT-2d (S221-S223), and EBT-8d (S281-S283) represent the SC205 (2×) treatment group at 0, 2, and 8 days mite infection, respectively. Samples SBT-0d (S401-S403), SBT-2d (S421-S423), and SBT-8d (S481-S483) represent the SC205 (4×) treatment group at 0, 2, and 8 days mite infection, respectively.

**
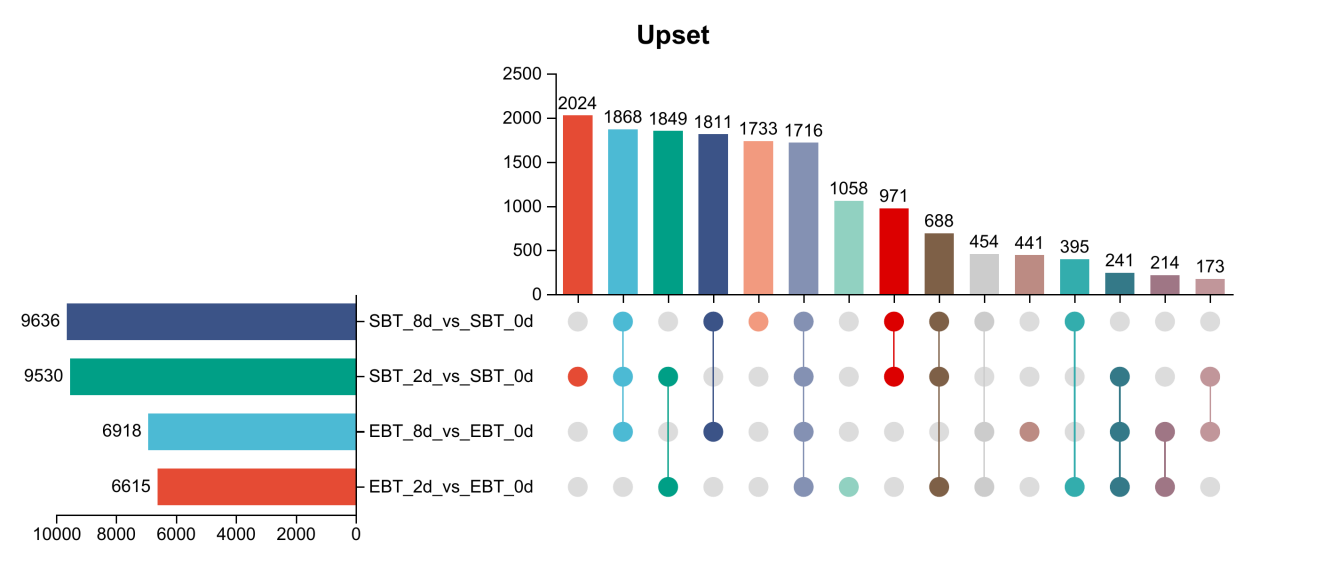
**

**Figure A2. Upset analysis of the transcriptome traits of different comparable groups.**


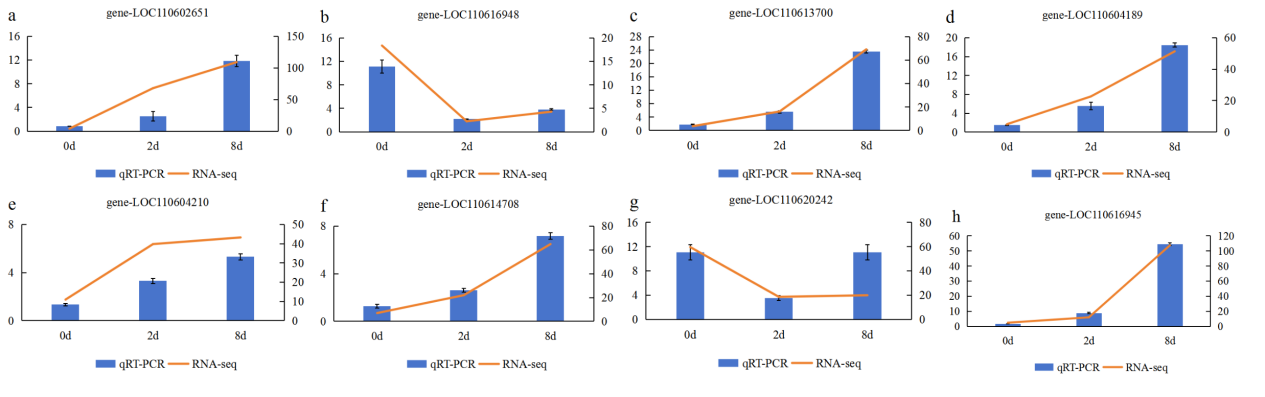


**
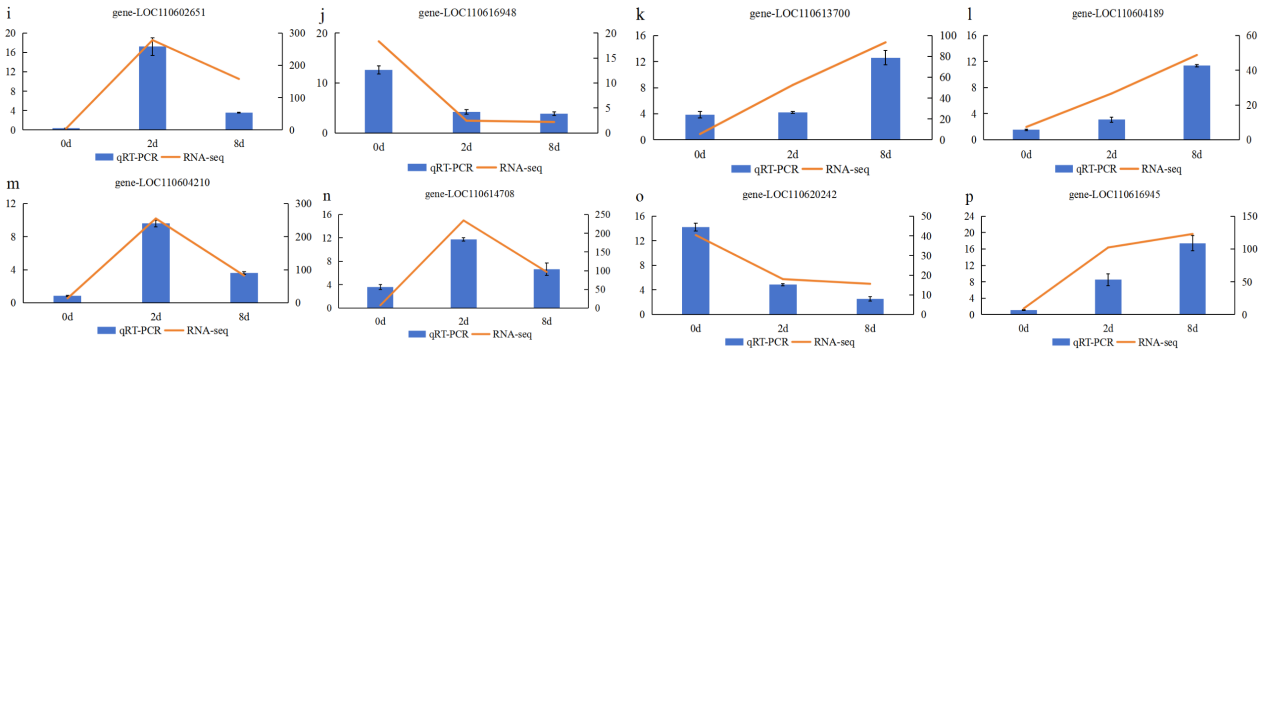
**

**Figure A3.** qRT-PCR verification of 8 genes related to common DEGs in four comparison groups, which diploid samples were graph a-h, autotetraploid samples were graph i-p. The Line chart expressing the expression level of the transcriptome (FPKM).

**Figure A4.** GO enrichment analysis of genotype-specific DEGs for (a) SC205 (2×) (2121 DEGs) and (b) SC205 (4×) (5036 DEGs) under 2-day pest hazards, respectively.


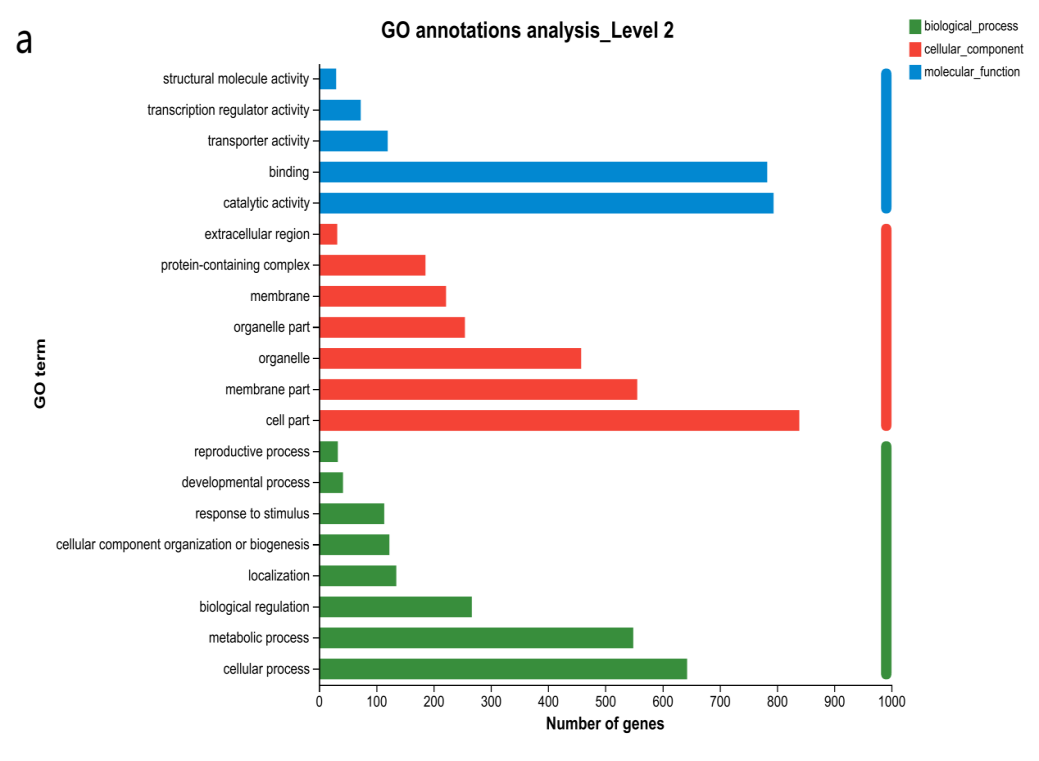

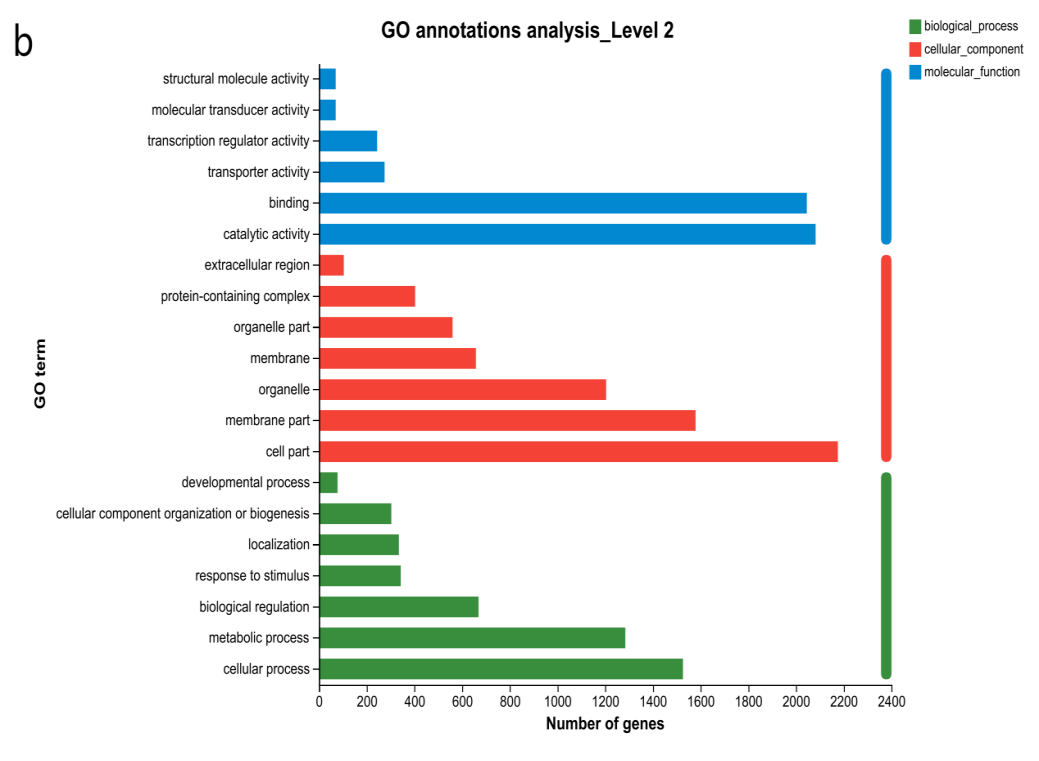


**Figure A5.** KEGG analysis of genotype-specific DEGs for (a) SC205 (2×) (2121 DEGs) and (b) SC205 (4×) (5036 DEGs) under 2-day pest hazards, respectively.


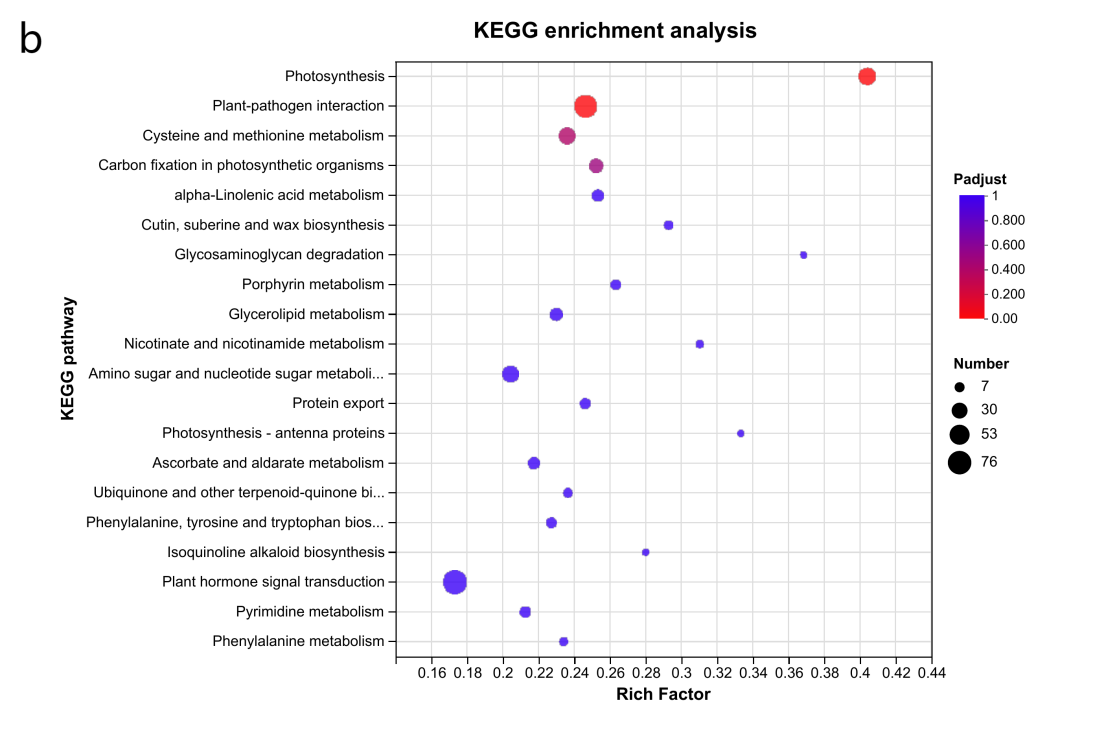

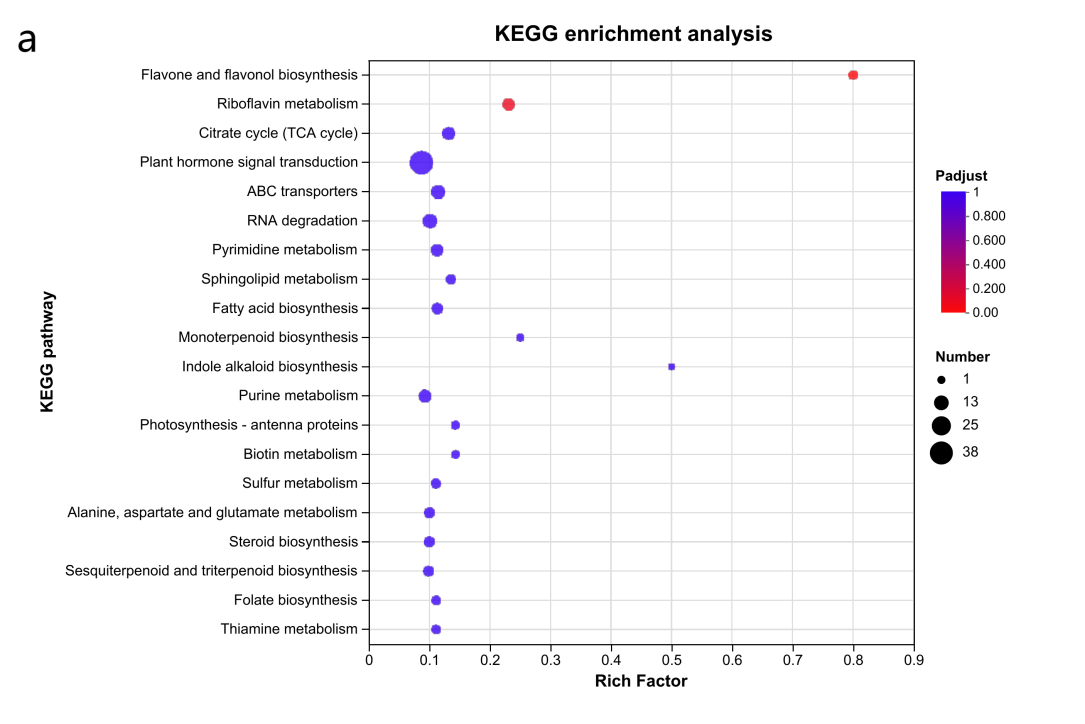


**Figure A6.** GO enrichment analysis of genotype-specific DEGs for (a) SC205 (2×) (1069 DEGs) and (b) SC205 (4×) (3787 DEGs) under 8-day pest hazards, respectively.


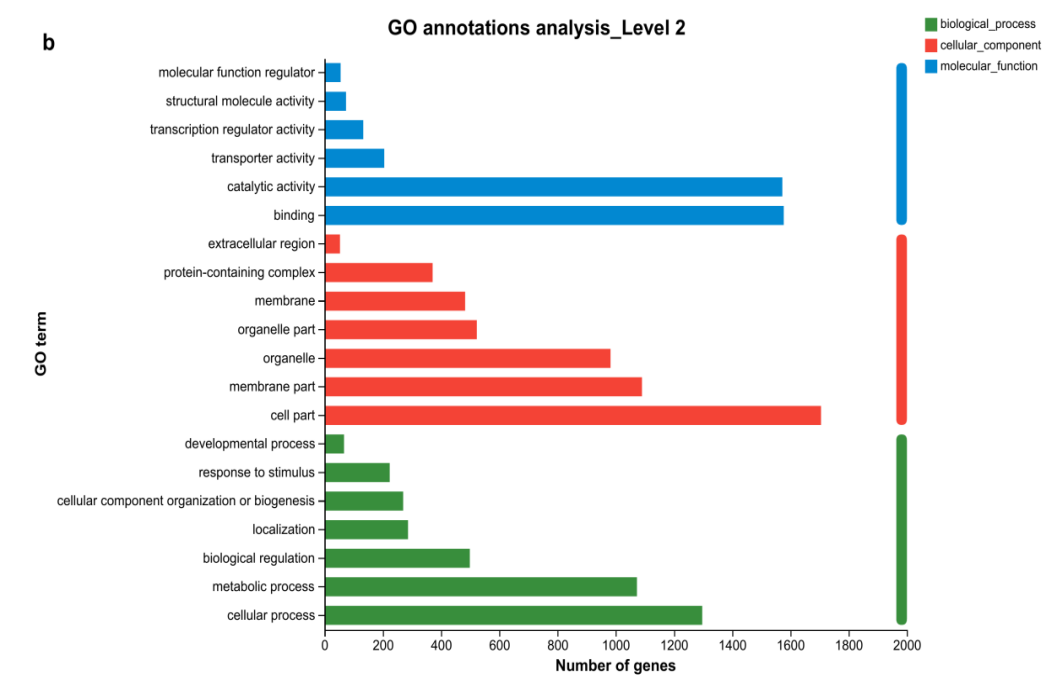

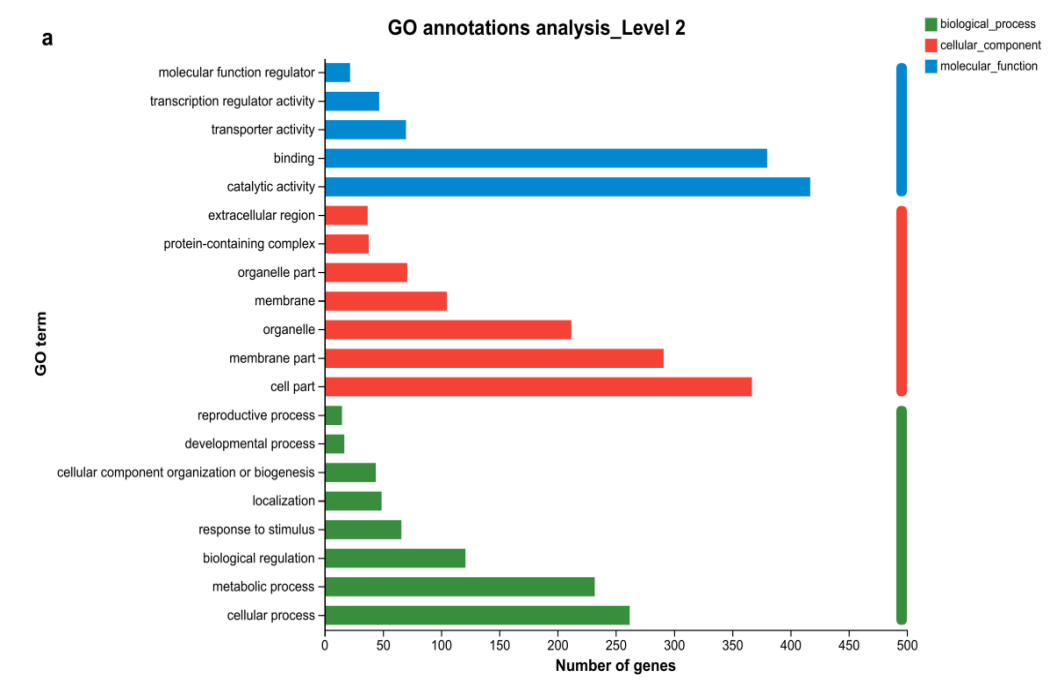


**Figure A7.** KEGG analysis of genotype-specific DEGs for (a) SC205 (2×) (1069 DEGs) and (b) SC205 (4×) (3787 DEGs) under 8-day pest hazards, respectively.


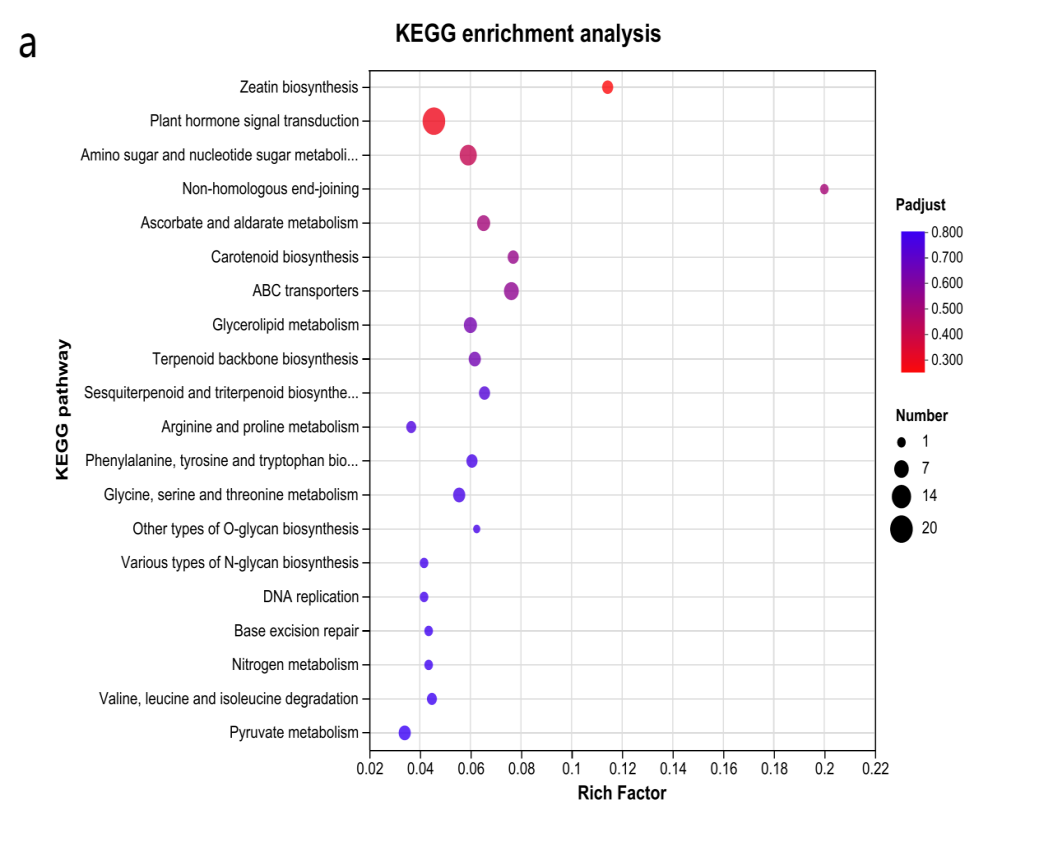

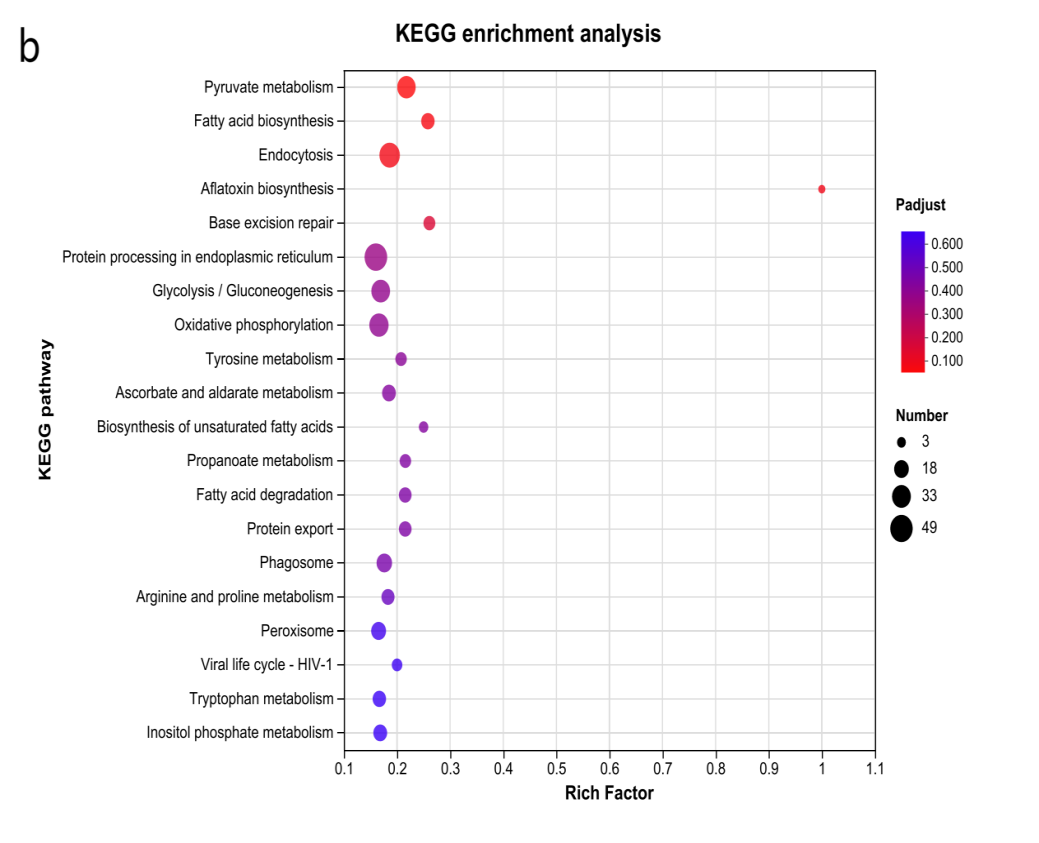


**
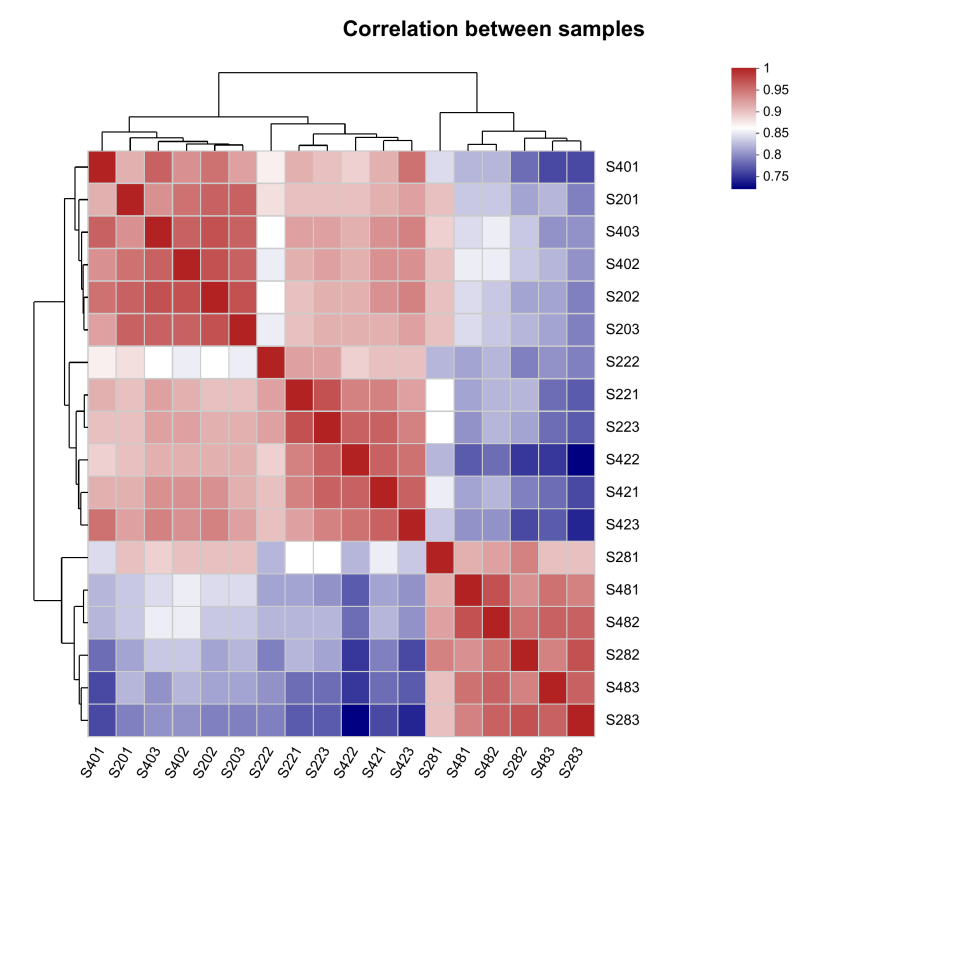
Figure A8.** Correlation between samples in the metabolome analysis.

Note: Samples EBT-0d (S201-S203), EBT-2d (S221-S223), and EBT-8d (S281-S283) represent the SC205 (2×) treatment group at 0, 2, and 8 days mite infection, respectively. Samples SBT-0d (S401-S403), SBT-2d (S421-S423), and SBT-8d (S481-S483) represent the SC205 (4×) treatment group at 0, 2, and 8 days mite infection, respectively.

**
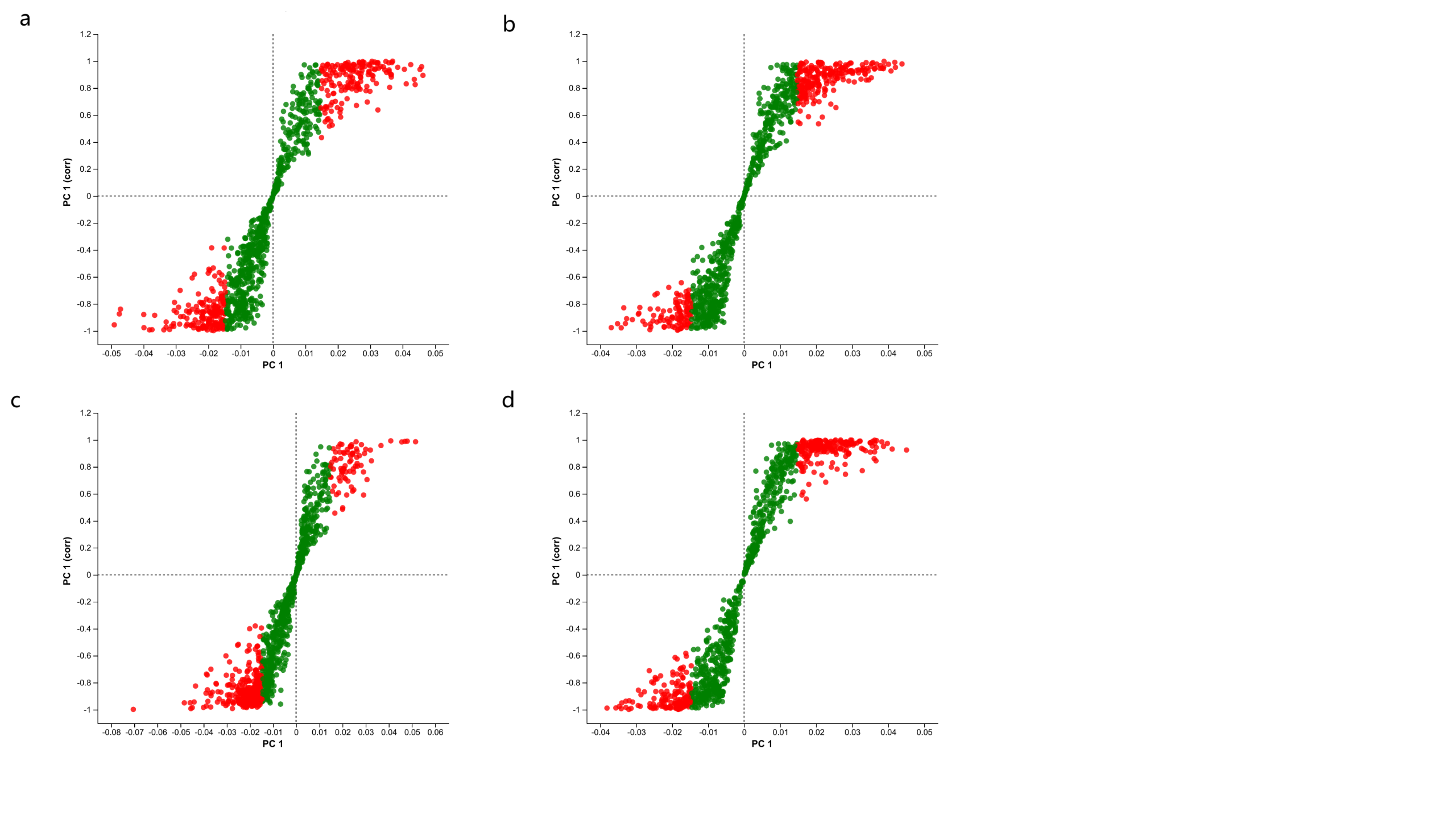
Figure A9.** OPLS-DAs analysis in the group of EBT_2d vs EBT_0d (a); EBT_8d vs EBT_0d (b); SBT_2d vs SBT_0d (c) and SBT_8d vs SBT_0d (d).

**Figure A10.** Volcano plot indicating upregulated and downregulated DAMs in the group of EBT_2d vs EBT_0d; EBT_8d vs EBT_0d; SBT_2d vs sBT_0d and SBT_8d vs SBT_0d.


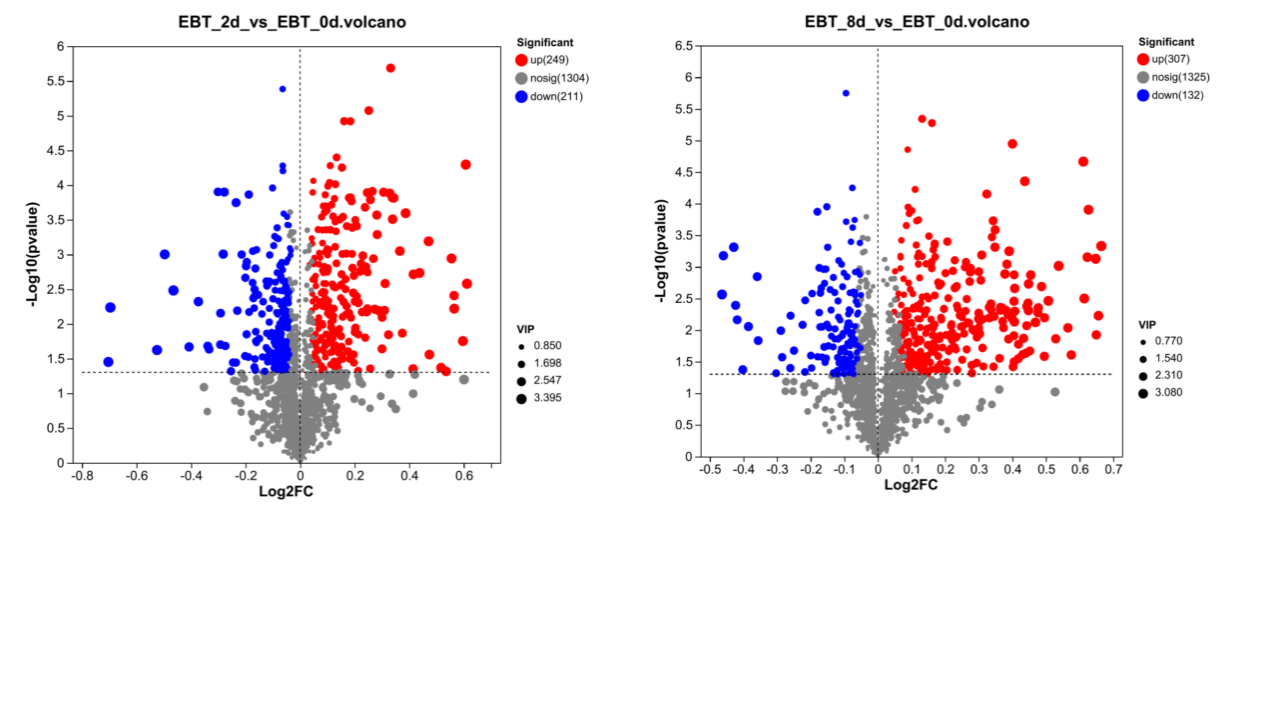

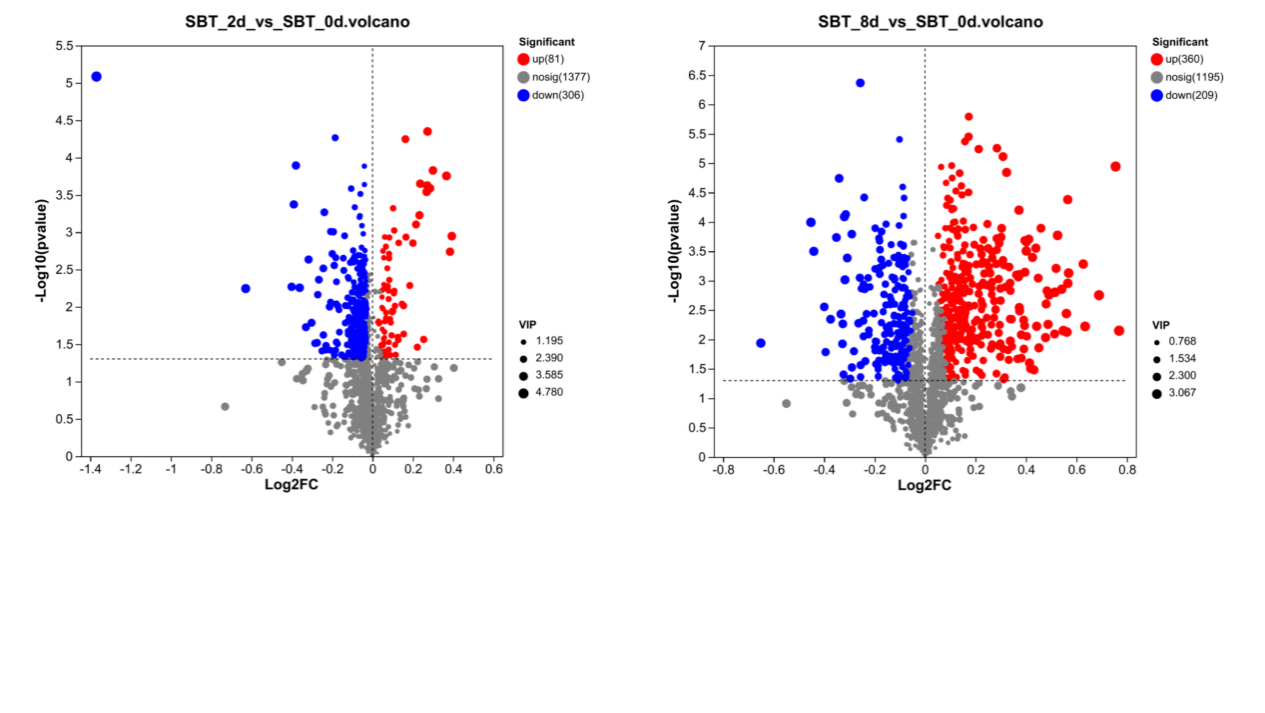


**Figure A11.** KEGG analysis in 279 specific DAMs of SC205 (2×)(a) and 206 specific DAMs of SC205 (4×)(b) in the 2 d *T. cinnabarinus* infection.


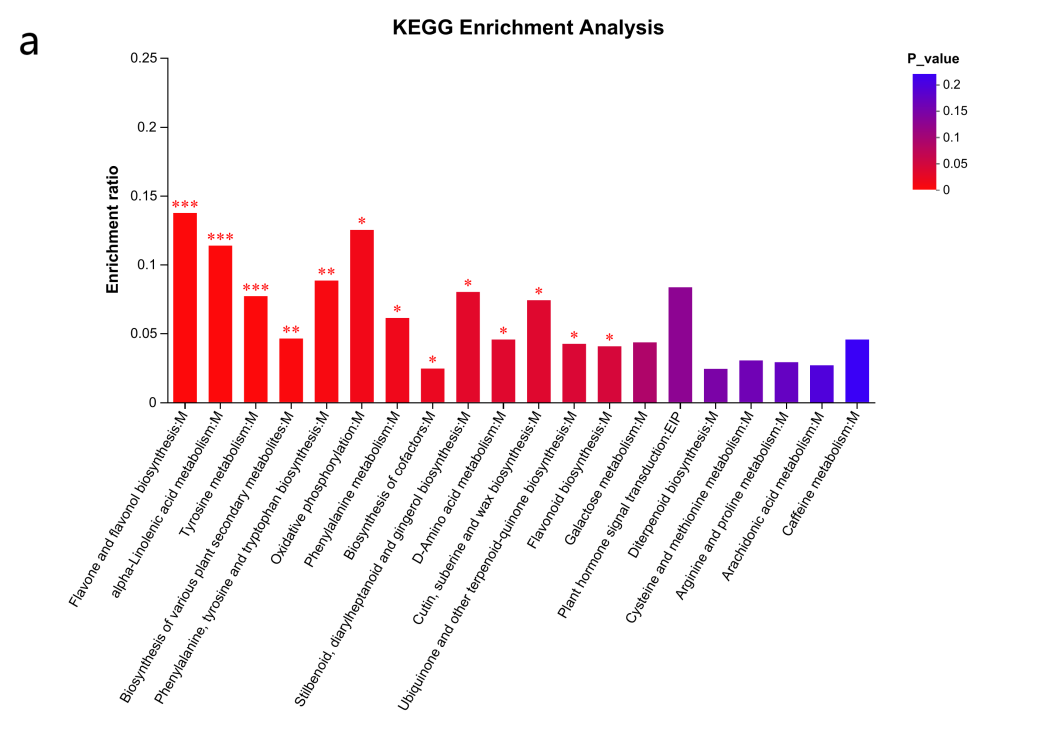

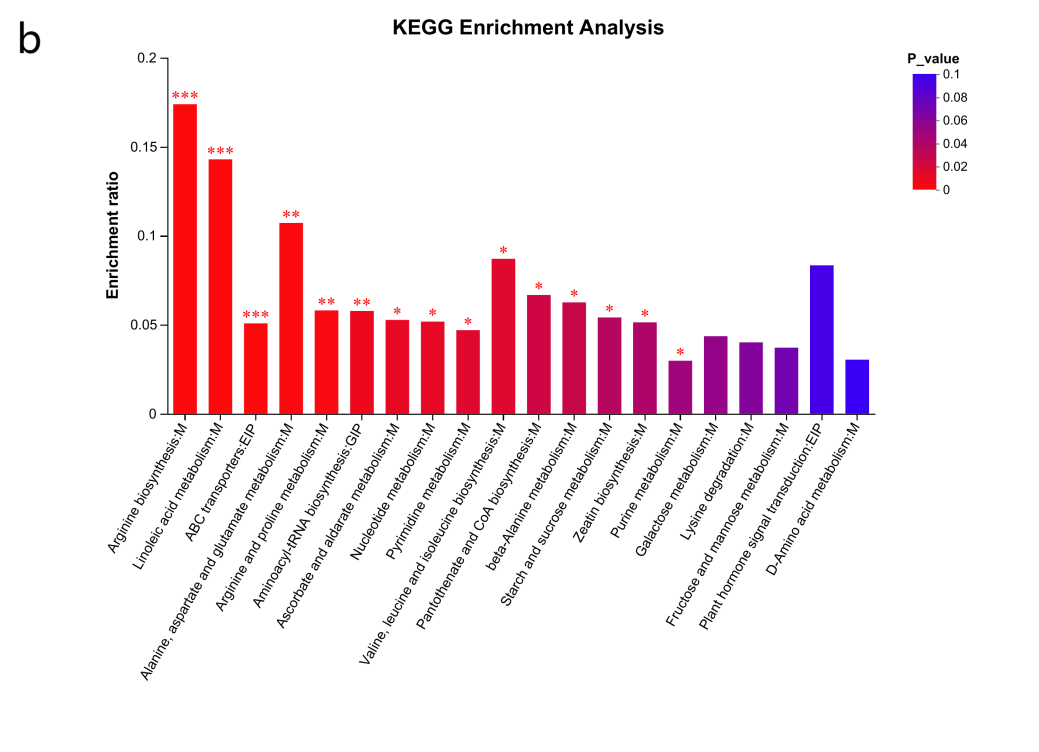


Note: * indicates the significant difference at 0.05 level, * * indicates the significant difference at 0.01 level, * * * indicates the significant difference at less than 0.01 level.

**Figure A12.** KEGG analysis in 96 specific DAMs of SC205 (2×)(a) and 226 specific DAMs of SC205 (4×)(b) in the 8 d *T. cinnabarinus* infection.


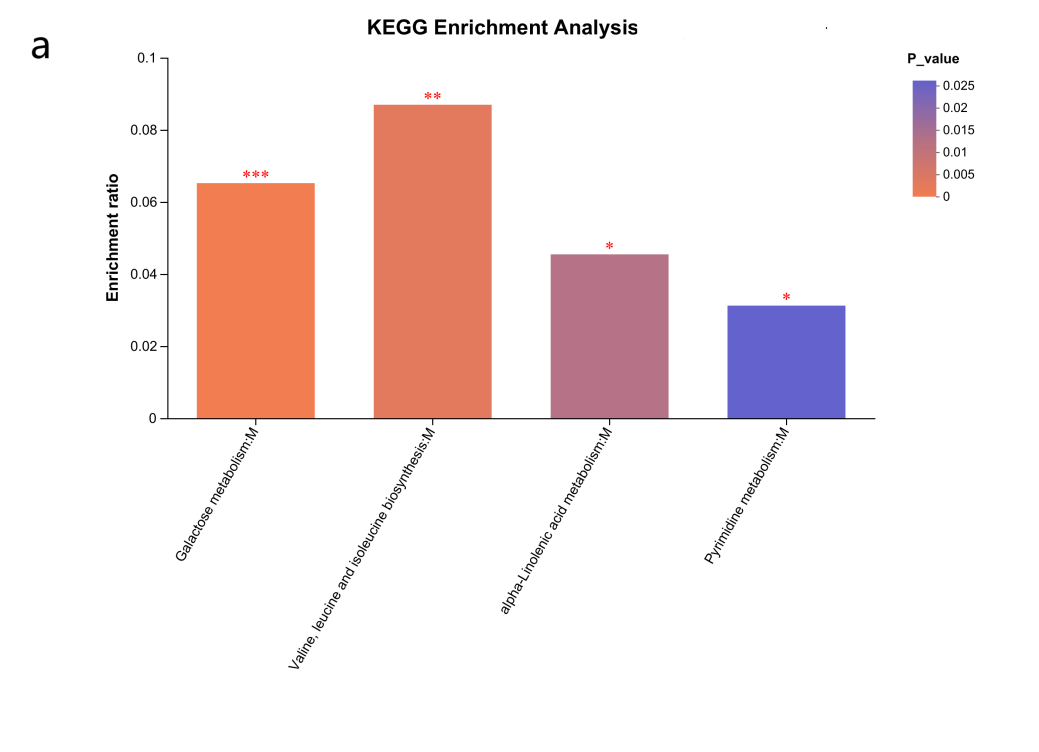

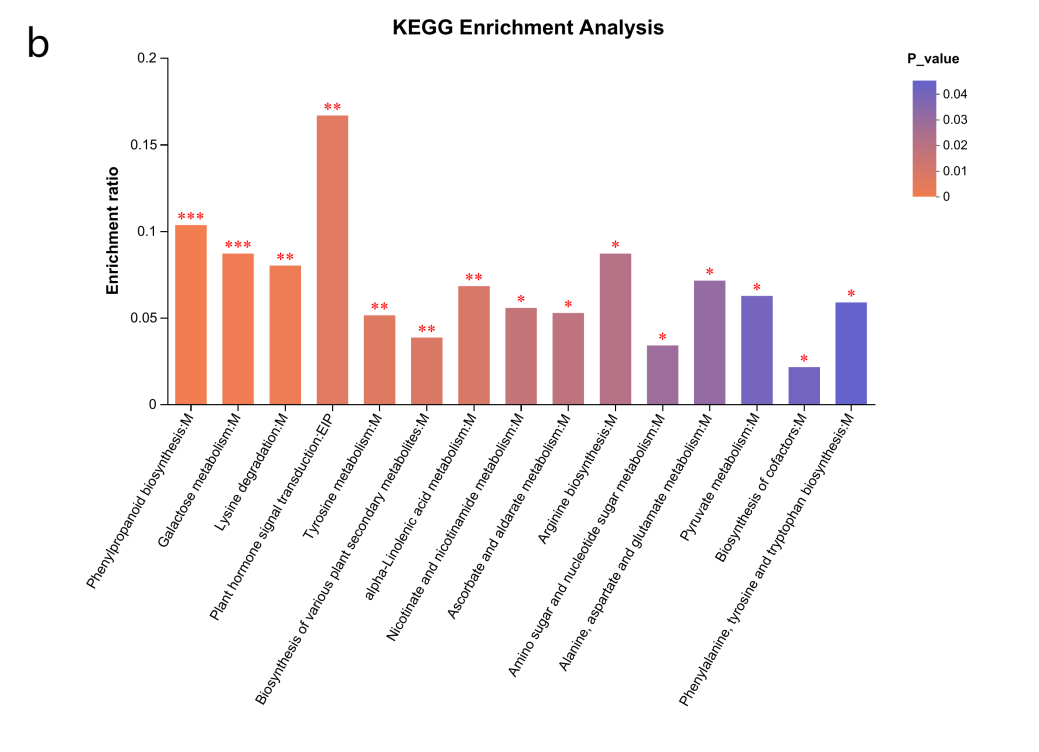


**Figure A13.** KEGG analysis of the metabolome and transcriptome in the 2d pest hazards groups (EBT_2d vs EBT_0d, a; SBT_2d vs SBT_0d, b).


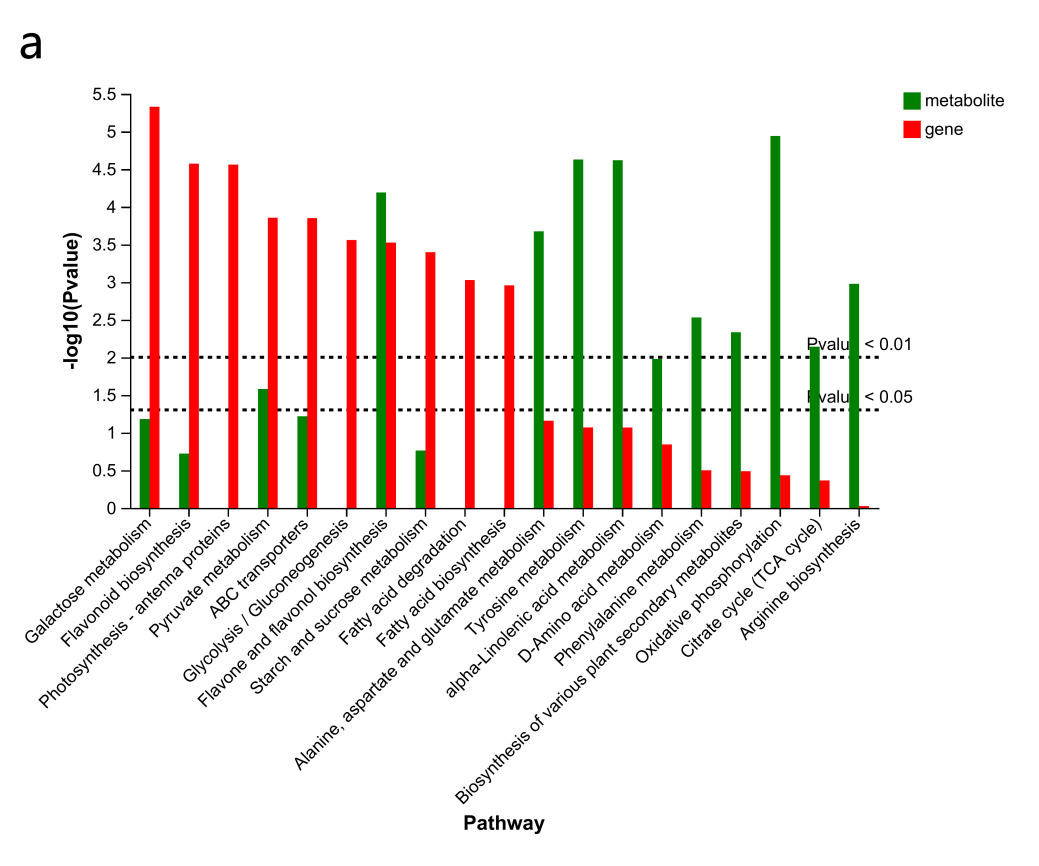

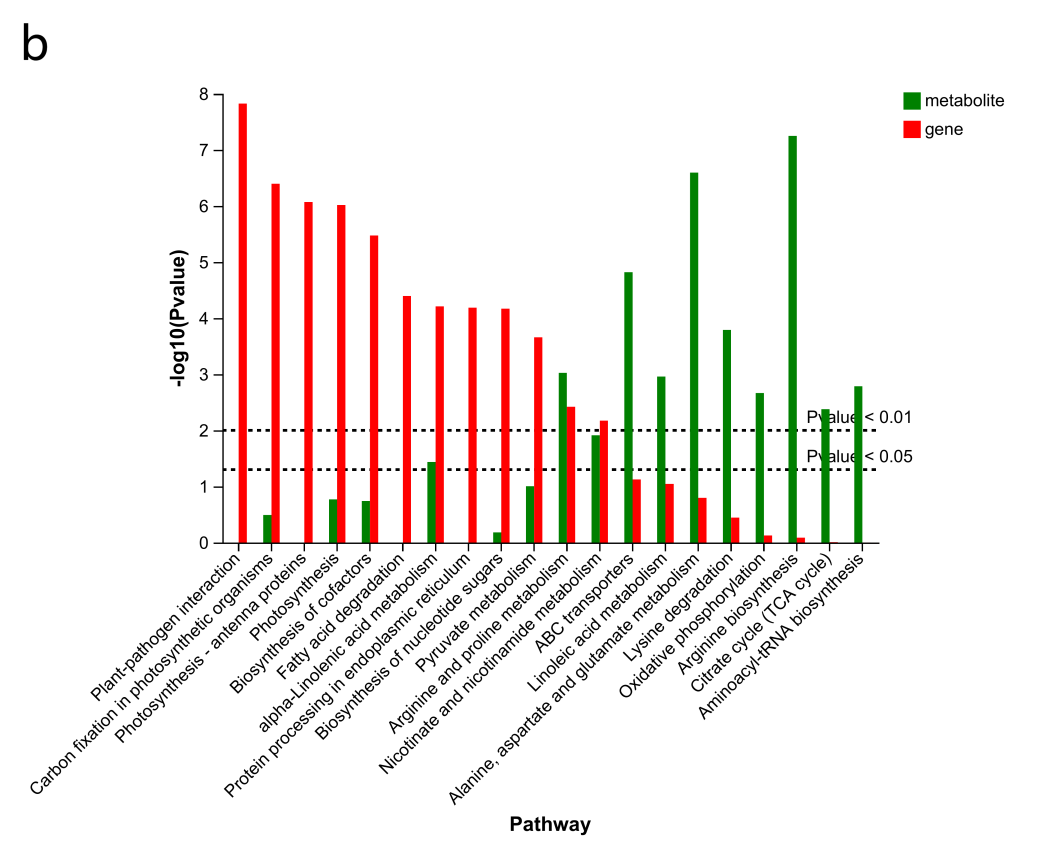

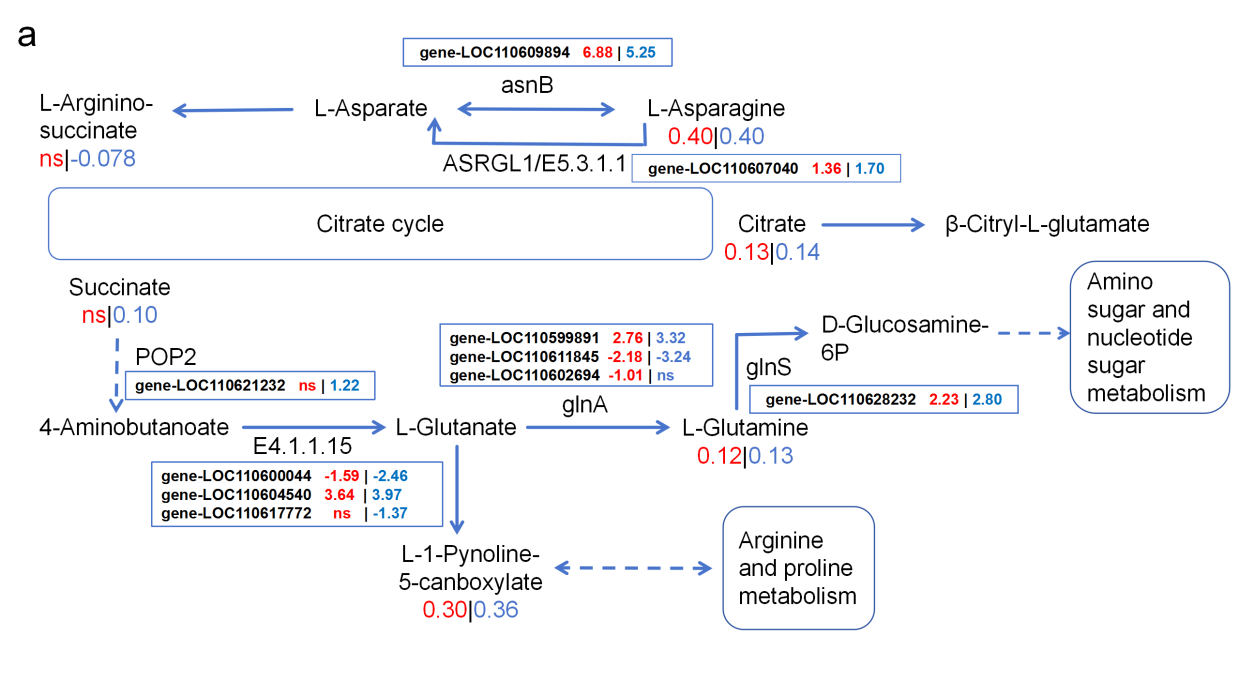

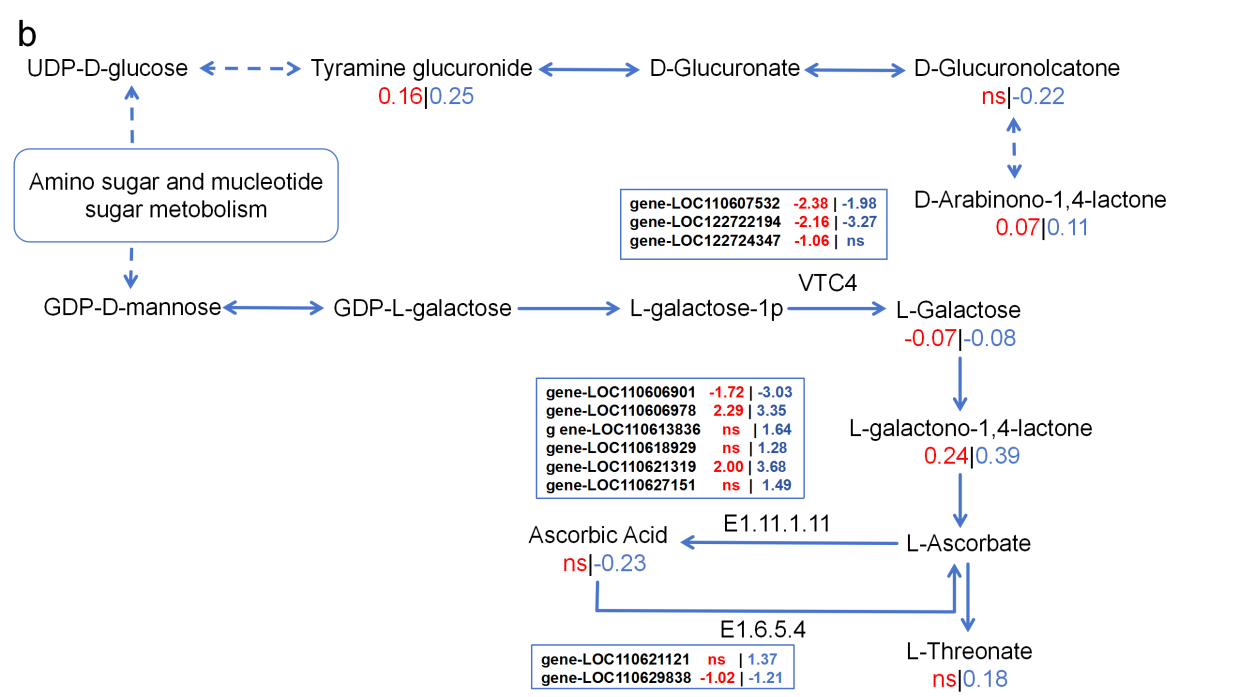

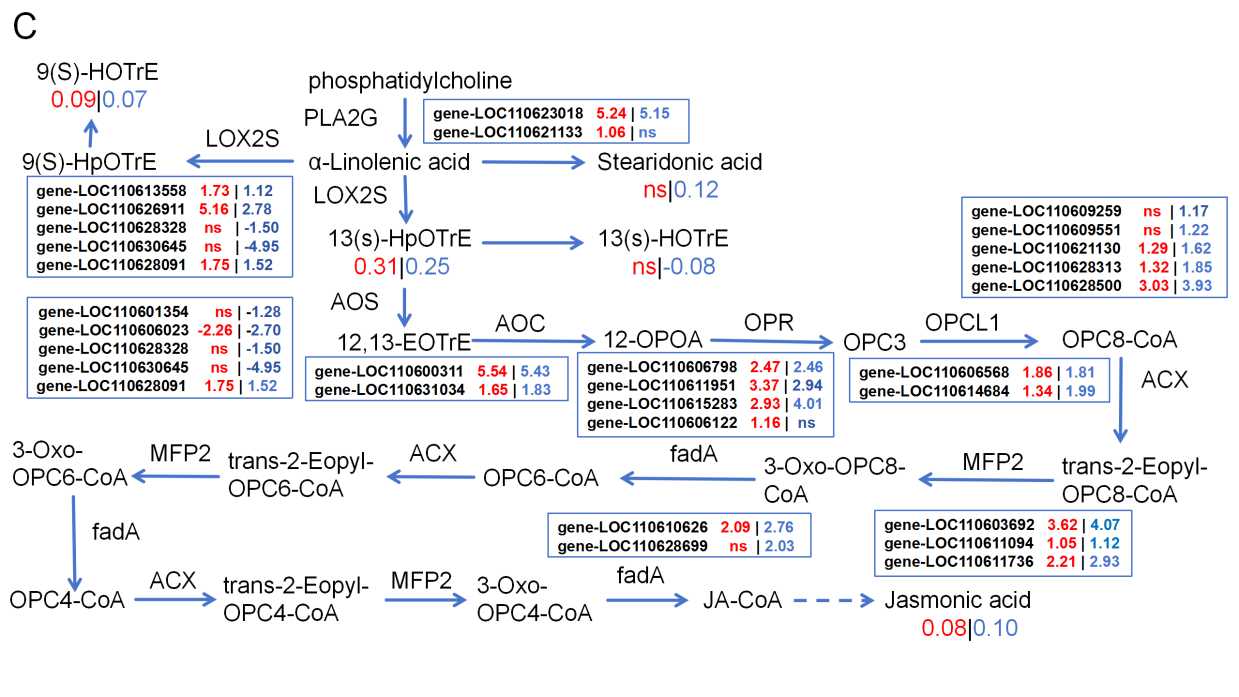


**Figure A14.** Network of the alanine, aspartate and glutamate metabolism pathway (a), ascorbate and aldarate metabolism pathway (b), and alpha-linolenic acid metabolism pathway (c) in the 8d pest hazards. The location of the node is metabolites and the substances on the line are enzymes. Some of them that were unaffected by pest hazards have been omitted from this graph. The red numbers represent the |log2fold change| of enzyme or metabolites for SC205 (2×), while the blue number represents the |log2fold change| of enzyme or metabolites for for SC205 (4×).

Note：ns，no significant.
